# Bayesian Additive Regression Trees for Group Testing Data

**DOI:** 10.1002/sim.70052

**Published:** 2025-03-14

**Authors:** Madeleine E. St. Ville, Christopher S. McMahan, Joe D. Bible, Joshua M. Tebbs, Christopher R. Bilder

**Affiliations:** ^1^ *Eunice Kennedy Shriver* National Institute of Child Health and Human Development, National Institutes of Health Bethesda MD USA; ^2^ School of Mathematical and Statistical Sciences Clemson University Clemson SC USA; ^3^ Department of Statistics University of South Carolina Columbia SC USA; ^4^ Department of Statistics University of Nebraska‐Lincoln Lincoln NE USA

**Keywords:** decision trees, latent variable modeling, machine learning, non‐parametric regression, pooled testing

## Abstract

When screening for low‐prevalence diseases, pooling specimens (e.g., blood, urine, swabs, etc.) through group testing has the potential to substantially reduce costs when compared to testing specimens individually. A common goal in group testing applications is to estimate the relationship between an individual's true disease status and their individual‐level covariate information. However, estimating such a relationship is a non‐trivial problem because true individual disease statuses are unknown due to the group testing protocol and the possibility of imperfect testing. While several regression methods have been developed in recent years to accommodate the complexity of group testing data, the functional form of covariate effects is typically assumed to be known. To avoid model misspecification and to provide a more flexible approach, we propose a Bayesian additive regression trees framework to model the individual‐level probability of disease with potentially misclassified group testing data. Our methods can be used to analyze data arising from any group testing protocol with the goal of estimating unknown functions of covariates and assay classification accuracy probabilities.

## 1 Introduction

When screening for infectious diseases, group testing has become a popular alternative to individual testing due to its cost‐effectiveness and efficiency. Fundamentally, in group testing one combines individual specimens (e.g., blood, urine, swabs, etc.) to form a pooled specimen that is tested for the presence of disease. In many group testing protocols, individuals contributing to a pooled specimen that tests negatively are classified as negative at the expense of just a single diagnostic assay; in contrast, positive pools are resolved through further testing. The infectious disease literature is replete with applications of group testing being used to detect HIV [1, 2], gonorrhea and chlamydia [3], influenza A/B [4], Zika [5], tuberculosis [6], and SARS‐CoV‐2 [7, 8].

A common task in disease surveillance is estimating the probability of disease for individuals and identifying associated risk factors. However, accurately estimating the relationship between an individual's disease status and their covariates from group testing data is a non‐trivial problem; the true individual responses are obscured by the group testing protocol, and testing responses are potentially misclassified due to imperfect testing. These complications notwithstanding, there has been substantial growth in the development of regression methods for group testing data. Existing research includes parametric approaches by Vansteelandt et al. [9], Huang and Tebbs [10], Chen et al. [11], and Delaigle and Tan [12], as well as non‐parametric approaches by Delaigle and Meister [13], Delaigle et al. [14], and Delaigle and Hall [15]. McMahan et al. [16] recently proposed a Bayesian approach within a generalized linear model (GLM) framework that boasts three strengths; namely, one can analyze data arising from any group testing protocol to include retesting information, one can incorporate historical information about disease prevalence, and the framework allows for the estimation of assay accuracy probabilities. McMahan et al. [16] also motivated the development of several modeling extensions [17, 18]. However, when viewed collectively, a limitation of many existing methods is that they require one to specify the functional form of the relationship between disease status and multiple covariates. This can be challenging to do when the relationship is complex. In particular, non‐linear and/or high‐order interaction effects are potentially ignored which can result in model misspecification and biased inference.

In this article, we propose a Bayesian additive regression trees (BART) modeling framework for group testing data. BART is an ensemble machine learning technique and is well‐equipped to handle large complex group testing data sets. It builds a series of decision trees that partition the input space into regions and make predictions based on covariate values within each region. It automatically captures complex high‐order interaction effects without requiring the researcher to specify anything about the functional form. Furthermore, BART extends the ensemble of decision trees by incorporating Bayesian modeling to quantify uncertainty in parameter estimates and regularize the fit. Our approach preserves the core strengths of McMahan et al. [16] while allowing for more flexible modeling. In turn, this provides a more accurate estimation and a better understanding of the relationship between disease status and covariates.

The remainder of this article is organized as follows. In Section 2, we introduce our proposed BART model and discuss assumptions. In Section 3, we describe the data augmentation steps that facilitate a Bayesian framework and provide details on posterior sampling. In Section 4, we present the results of simulation studies to assess performance under a variety of settings for different group testing protocols. In Section 5, we use our methods to analyze a chlamydia group testing data set collected at a public health laboratory in Iowa. In Section 6, we conclude with a summary discussion. Additional technical details, simulation evidence, and data analysis results are shown in the Supporting Information.

## 2 Notation and Model

Suppose a group testing protocol is used to test N individuals for a binary characteristic, such as the presence or absence of a disease. Let ${\tilde{Y}}_{i}$, for i=1,…,N, denote the true disease status of the ith individual, with the usual convention that ${\tilde{Y}}_{i}=1$ if the individual is truly positive, ${\tilde{Y}}_{i}=0$ otherwise. Let $x_{i}={\left(x_{i1},\ldots ,x_{iQ}\right)}^{\prime }$ denote a vector of Q covariates observed for the ith individual. We aggregate the individuals' true disease statuses into the vector $\tilde{Y}={\left({\tilde{Y}}_{1},\ldots ,{\tilde{Y}}_{N}\right)}^{\prime }$ and their covariates into the matrix $X=(x_{1}\cdots \;x_{N})$. For modeling purposes, we assume the individuals' true disease statuses are conditionally independent given their individual‐level covariates and that the relationship between ${\tilde{Y}}_{i}$ and $x_{i}$ is

(1) $$\Phi^{-1}\{P({\tilde{Y}}_{i}=1\;|\;x_{i})\}=f(x_{i})$$

where $\Phi^{-1}(\cdot )$ is the inverse cumulative distribution function of a standard normal random variable (i.e., the probit link function) and f(·) is an unknown function, regarded as an infinite‐dimensional parameter. To reduce its dimension while also maintaining adequate modeling flexibility, we approximate f(·) by using additive regression trees; i.e., we approximate f(·) by an ensemble of K regression trees in the following manner:

(2) $$f(x_{i})\approx \eta (x_{i}):=\overset{K}{\underset{k=1}{\sum }}g(x_{i};T_{k},M_{k})$$

where $T_{k}$ is the kth regression tree structure consisting of a set of interior node decision rules and a set of $b_{k}$ terminal nodes and $M_{k}={\left(\mu_{k1},\ldots ,\mu_{kb_{k}}\right)}^{\prime }$ is a $b_{k}$‐dimensional vector of terminal node parameters. The function $g(x_{i};T_{k},M_{k})$ returns the value $\mu_{kt}\in M_{k}$ if $x_{i}$ is assigned to the tth terminal node based on the interior node decision rules of $T_{k}$. The decision rules provide information regarding which covariate to split on and the associated cutoff value. These are binary splits based on a single covariate and are of the form $\left\{x_{iq}\leq c\right\}$ vs. $\left\{x_{iq}>c\right\}$ for a cutoff value c. Taken together, $g(x_{i};T_{k},M_{k})$ can be viewed as a multi‐dimensional step function that can aptly account for many features, including non‐linear effects and interactions of varying orders. Note that in (2), K is the (typically fixed) number of regression trees. Setting K to be large is recommended for flexible estimation, for example, Chipman et al. [19] showed the default K=200 yields good predictive performance.

**FIGURE 1:**
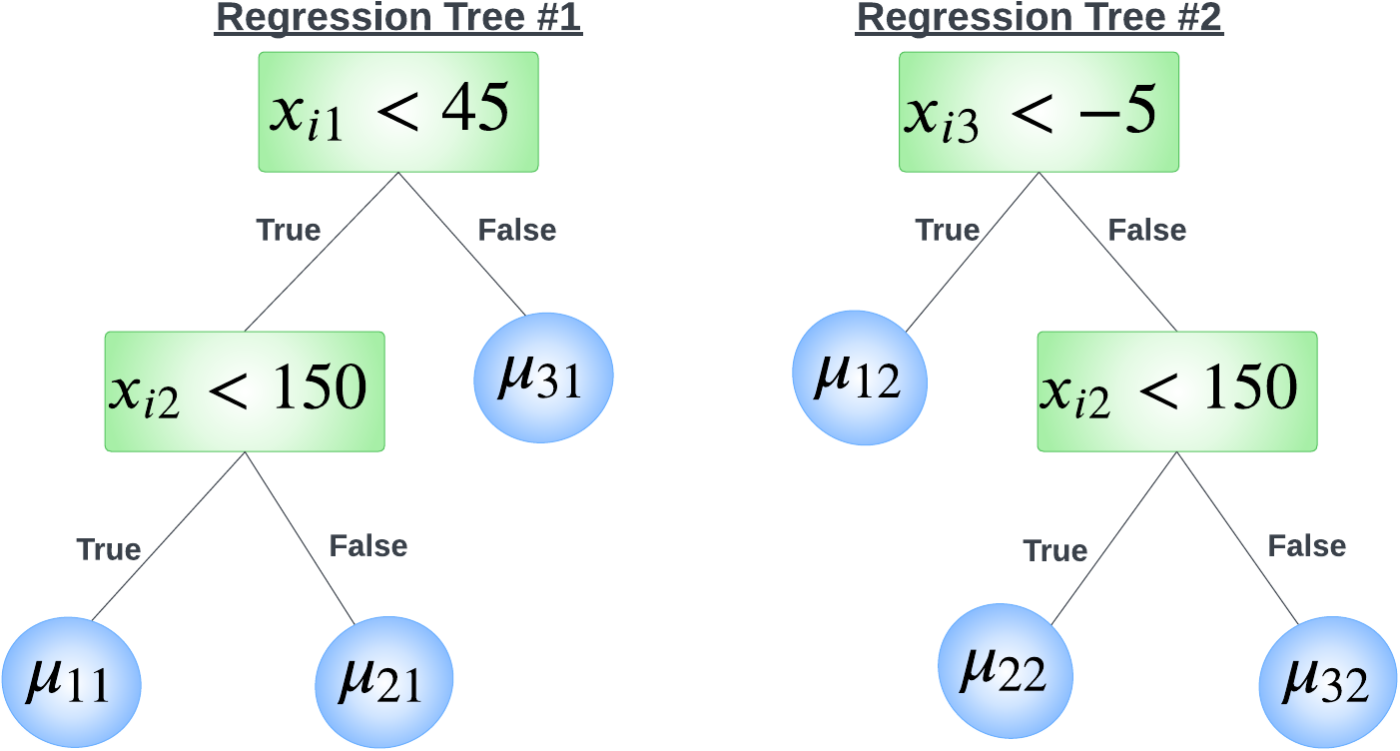
Sum of regression trees illustration using two trees. Each tree uses two predictors. The tree on the left uses $x_{i1}$ and $x_{i2}$. The tree on the right uses $x_{i2}$ and $x_{i3}$. Each tree has three terminal nodes.

For readers unfamiliar with the sum‐of‐trees model, consider the following simple example with an ensemble of K=2 trees and Q=3 covariates. Suppose we are given the two trees in Figure 1. Each tree uses two predictors to split the data into subgroups; the first tree (k=1) on the left in Figure 1 uses $x_{i1}$ and $x_{i2}$, while the second tree (k=2) on the right uses $x_{i2}$ and $x_{i3}$. For each tree, each value of $x_{i}$ is assigned to a single terminal node by following a sequence of decision rules at each interior node from top to bottom, where it is finally assigned a parameter value associated with that terminal node. For the hypothetical data on 5 individuals given in Table 1, one can see the quantity being “summed” in the final model for the ith individual is the terminal node parameter each tree structure assigns to this individual. Tan and Roy [20] provide additional regression tree examples in their excellent expository review of BART and its associated models.

**TABLE 1:** Terminal nodes assigned to five individuals (hypothetical data) for the K=2 regression trees in Figure 1. Values of $\sum_{k=1}^{2}g(x_{i};T_{k},M_{k})$ are also shown.

i	$x_{i1}$	$x_{i2}$	$x_{i3}$	$g(x_{i};T_{1},M_{1})$	$g(x_{i};T_{2},M_{2})$	$\sum_{k=1}^{2}g(x_{i};T_{k},M_{k})$
1	56	110	−13	$\mu_{31}$	$\mu_{12}$	$\mu_{31}+\mu_{12}$
2	27	173	−3	$\mu_{21}$	$\mu_{32}$	$\mu_{21}+\mu_{32}$
3	41	94	5	$\mu_{11}$	$\mu_{22}$	$\mu_{11}+\mu_{22}$
4	30	213	−9	$\mu_{21}$	$\mu_{12}$	$\mu_{21}+\mu_{12}$
5	48	168	39	$\mu_{31}$	$\mu_{32}$	$\mu_{31}+\mu_{32}$

BART casts the sum‐of‐trees model into a Bayesian paradigm and controls the size and effect of individual trees by imposing regularization priors [19]. Of course, if individuals' true disease statuses were observed, one could estimate the BART model using existing R packages; e.g., BayesTree [19], bartMachine [21], BART [22], etc. However, due to the effects of both pooling and imperfect testing, the individuals' true disease statuses are unobserved in group testing. Instead, the observed data available for model fitting consist of possibly error‐contaminated test responses that are measured on pools and/or individuals according to the group testing protocol in use. Further complicating the data structure, many protocols require individuals to be tested in multiple pools including pools which may overlap with others [1, 23].

To maintain generality and to accommodate data from any group testing protocol, we track pool membership through the index sets ${𝒫}_{j}\subset \{1,2,\ldots ,N\}$, where ${𝒫}_{j}$ consists of the indices of individuals who contributed to the jth pool, j=1,…,J. Let $Z_{j}$ denote the test outcome from assaying the jth pool, where $Z_{j}=1$ if the pool tests positively and $Z_{j}=0$ otherwise. The sensitivity and specificity of the assay used to test the jth pool are $S_{ej}=P(Z_{j}=1|\;{\tilde{Z}}_{j}=1)$ and $S_{pj}=P(Z_{j}=0|\;{\tilde{Z}}_{j}=0)$, respectively, where ${\tilde{Z}}_{j}$ is the true disease status of the jth pool, that is, ${\tilde{Z}}_{j}=I(\sum_{i\in {𝒫}_{j}}{\tilde{Y}}_{i}>0)$, where I(·) is the indicator function. In some settings, it may be reasonable to assume $S_{ej}$ and $S_{pj}$ are known a priori. If not, one can regard these as unknown parameters and estimate them following the approach outlined in McMahan et al. [16]. That is, one can divide the test outcomes into L strata created by factors related to assay characteristics and performance (e.g., pool size, type of assay used, specimen type, etc.) and define the index set ℳ(l)={j:thejth test outcome is in thelth stratum}. We assume assay accuracy probabilities are constant within each stratum but potentially vary among the L strata. Define $S_{e(l)}$ and $S_{p(l)}$ to be the sensitivity and specificity of the assay associated with the lth stratum, respectively, that is, $S_{ej}=S_{e(l)}$ and $S_{pj}=S_{p(l)}$ if and only if j∈ℳ(l). Examples of how strata might be created in practice are shown in Sections 4 and 5.

The conditional distribution of $Z={\left(Z_{1},\ldots ,Z_{J}\right)}^{\prime }$ can be expressed as

(3) $$\begin{array}{ll} & \pi (Z\;|\;S_{e},S_{p},X,T,M)\\ & \;=\underset{\tilde{Y}\in {\left\{0,1\right\}}^{N}}{\sum }\left[\overset{L}{\underset{l=1}{\prod }}\underset{j\in ℳ(l)}{\prod }{\left\{S_{e(l)}^{Z_{j}}{\left(1-S_{e(l)}\right)}^{1-Z_{j}}\right\}}^{{\tilde{Z}}_{j}}\right.\\ & \;\left.{\left\{{\left(1-S_{p(l)}\right)}^{Z_{j}}S_{p(l)}^{1-Z_{j}}\right\}}^{1-{\tilde{Z}}_{j}}\overset{N}{\underset{i=1}{\prod }}{\left\{\Phi (\eta_{i})\right\}}^{{\tilde{Y}}_{i}}{\left\{1-\Phi (\eta_{i})\right\}}^{1-{\tilde{Y}}_{i}}\right] \end{array}$$

where $S_{e}={\left(S_{e(1)},\ldots ,S_{e(L)}\right)}^{\prime }$, $S_{p}={\left(S_{p(1)},\ldots ,S_{p(L)}\right)}^{\prime }$, T is the collection of regression trees $T_{1},\ldots ,T_{K}$, $M={\left(M_{1}^{\prime },\ldots ,M_{K}^{\prime }\right)}^{\prime }$, and $\eta_{i}=\eta (x_{i})$. To derive (3), we assume the observed testing responses in Z are conditionally independent given their true statuses $\tilde{Z}={\left({\tilde{Z}}_{1},\ldots ,{\tilde{Z}}_{J}\right)}^{\prime }$ and $Z|\tilde{Z}$ does not depend on the covariates in X. These assumptions are common in the group testing literature for regression; see, e.g., Vansteelandt et al. [9] and Xie [24], and are reasonable when misclassification is driven primarily by factors related to test implementation. Evaluating the data model in (3) requires taking the sum over ${\left\{0,1\right\}}^{N}$, which denotes the collection of all $2^{N}$ possible realizations of $\tilde{Y}$. For this reason, evaluating (3) can be computationally burdensome at best and completely intractable at worst. We make use of a data augmentation strategy, described in Section 3.1, to develop a posterior sampling algorithm that circumvents the need to directly evaluate this data model. This posterior algorithm operates in the same way regardless of which group testing protocol is used to collect the data.

To complete our Bayesian model, we specify priors for each of the unknown model parameters; i.e., the parameters governing the sum‐of‐trees model in (2) and the assay accuracy probabilities. Recall the sum‐of‐trees model is determined by K trees and the corresponding terminal node parameters, that is, $\left(T_{1},M_{1}),\ldots ,(T_{K},M_{K}\right)$. Thus, we must impose priors on the kth tree structure, $T_{k}$, and the terminal node parameters given this kth tree structure, $M_{k}|T_{k}$, for each k=1,…,K. Assuming $\left(T_{1},M_{1}),\ldots ,(T_{K},M_{K}\right)$ are independent, we can write the prior distribution as

$$\begin{array}{ll} & \pi \{(T_{1},M_{1}),\ldots ,(T_{K},M_{K})\}\\ & \;=\overset{K}{\underset{k=1}{\prod }}\pi (T_{k},M_{k})=\overset{K}{\underset{k=1}{\prod }}\pi (M_{k}|T_{k})\pi (T_{k})=\overset{K}{\underset{k=1}{\prod }}\overset{b_{k}}{\underset{t=1}{\prod }}\pi (\mu_{kt}|T_{k})\pi (T_{k}) \end{array}$$

noting the last equality follows from the assumption that terminal node parameters are conditionally independent given their tree structure. To elicit the priors $\pi (T_{k})$ and $\pi (\mu_{kt}|T_{k})$, we follow the approach in Chipman et al. [19], which we now describe. We first specify $\pi (T_{k})$, the prior distribution on the kth tree structure, based on three probabilistic rules that control the size (i.e., number of terminal nodes) of the tree, the variables on which to split, and the locations of the split. The size of the tree is based on the depth of the terminal nodes, where a node at depth d∈{0,1,2,…} is non‐terminal (i.e., an interior node) with probability $\alpha {\left(1+d\right)}^{-\beta }$, where α∈(0,1) and β∈[0,∞). Default values of the hyperparameters recommended by Chipman et al. [19], α=0.95 and β=2, are used. These values tend to a priori favor smaller trees, for example, trees having 2 to 3 terminal nodes. For non‐terminal nodes, the variable on which to split is randomly selected from the set of available covariates, and the location of the split given the selected variable is sampled at random from the set of observed values for that variable. The prior for the terminal node parameters is $\mu_{kt}∼N(0,\sigma_{\mu }^{2})$, where $\sigma_{\mu }=3.0/\left(H\sqrt{K}\right)$. Chipman et al. [19] recommend H=2 as the default hyperparameter value, which we also adopt. The aim of this prior is to provide model regularization; it has the ability to shrink terminal node parameters thereby limiting the effect of the individual tree components. Finally, to acknowledge uncertainty in the assay accuracy probabilities, we specify independent beta priors $S_{e(l)}∼\text{beta}(a_{e(l)},b_{e(l)})$ and $S_{p(l)}∼\text{beta}(a_{p(l)},b_{p(l)})$, for l=1,…,L. When historical information about assay performance is available (e.g., from pilot studies used to validate the assay, etc.), one can incorporate it into the model by choosing hyperparameter values to reflect this prior knowledge. In the absence of any prior information, one can select $a_{e(l)}=b_{e(l)}=a_{p(l)}=b_{p(l)}=1$ corresponding to uniform priors.

## 3 Posterior Inference

### 3.1 Data Augmentation

To facilitate the development of an efficient posterior sampling algorithm and to avoid direct evaluation of (3), we propose a two‐stage data augmentation procedure. In the first stage, we introduce the individuals' true disease statuses ${\tilde{Y}}_{i}$ as latent random variables. This leads to the following joint conditional distribution:

$$\begin{array}{ll} & \pi (Z,\tilde{Y}\;|\;S_{e},S_{p},X,T,M)\\ & \;=\overset{L}{\underset{l=1}{\prod }}\underset{j\in ℳ(l)}{\prod }{\left\{S_{e(l)}^{Z_{j}}{\left(1-S_{e(l)}\right)}^{1-Z_{j}}\right\}}^{{\tilde{Z}}_{j}}{\left\{{\left(1-S_{p(l)}\right)}^{Z_{j}}S_{p(l)}^{1-Z_{j}}\right\}}^{1-{\tilde{Z}}_{j}}\\ & \;\overset{N}{\underset{i=1}{\prod }}{\left\{\Phi (\eta_{i})\right\}}^{{\tilde{Y}}_{i}}{\left\{1-\Phi (\eta_{i})\right\}}^{1-{\tilde{Y}}_{i}} \end{array}$$

Making use of the fact our model employs the probit link function, the second stage introduces a carefully constructed latent random variable $\omega_{i}$ for each i=1,…,N. These random variables independently follow a standard normal distribution such that $\omega_{i}>0$ if ${\tilde{Y}}_{i}=1$ and $\omega_{i}\leq 0$ if ${\tilde{Y}}_{i}=0$; see Albert and Chib [25]. This stage yields the following augmented likelihood:

(4) $$\begin{array}{ll} & \pi (Z,\tilde{Y},\omega \;|\;S_{e},S_{p},X,T,M)\\ & \;=\overset{L}{\underset{l=1}{\prod }}\underset{j\in ℳ(l)}{\prod }{\left\{S_{e(l)}^{Z_{j}}{\left(1-S_{e(l)}\right)}^{1-Z_{j}}\right\}}^{{\tilde{Z}}_{j}}{\left\{{\left(1-S_{p(l)}\right)}^{Z_{j}}S_{p(l)}^{1-Z_{j}}\right\}}^{1-{\tilde{Z}}_{j}}\\ & \;\times \overset{N}{\underset{i=1}{\prod }}\phi (\omega_{i}-\eta_{i})\left\{I({\tilde{Y}}_{i}=1,\omega_{i}>0)+I({\tilde{Y}}_{i}=0,\omega_{i}\leq 0)\right\} \end{array}$$

where $\omega ={\left(\omega_{1},\ldots ,\omega_{N}\right)}^{\prime }$ and ϕ(·) denotes the standard normal probability density function. These two stages of augmentation, together with our prior specifications, allow for the construction of a full Gibbs sampling algorithm for posterior inference.

### 3.2 Posterior Sampling

We briefly describe the full conditional distributions used in our posterior sampling algorithm. A complete description of the algorithm is in Appendix A of the Supporting Information. From (4), one can show the full conditional of ${\tilde{Y}}_{i}$ is ${\tilde{Y}}_{i}\;|\;Z,{\tilde{Y}}_{-i},S_{e},S_{p},T,M∼\text{Bernoulli}\{p_{i1}^{*}/(p_{i0}^{*}+p_{i1}^{*})\}$, where ${\tilde{Y}}_{-i}$ is the vector $\tilde{Y}$ with the ith element removed,

$$\begin{array}{ll} p_{i1}^{*} & =\Phi (\eta_{i})\overset{L}{\underset{l=1}{\prod }}\underset{j\in {ℐ}_{i}(l)}{\prod }S_{e(l)}^{Z_{j}}{\left(1-S_{e(l)}\right)}^{1-Z_{j}}\\ p_{i0}^{*} & =\left\{1-\Phi (\eta_{i})\right\}\overset{L}{\underset{l=1}{\prod }}\underset{j\in {ℐ}_{i}(l)}{\prod }{\left\{S_{e(l)}^{Z_{j}}{\left(1-S_{e(l)}\right)}^{1-Z_{j}}\right\}}^{I(s_{ij}>0)}\\ & \;{\left\{{\left(1-S_{p(l)}\right)}^{Z_{j}}S_{p(l)}^{1-Z_{j}}\right\}}^{I(s_{ij}=0)} \end{array}$$

$s_{ij}=\sum_{i^{\prime }\in {𝒫}_{j}:i^{\prime }\neq i}{\tilde{Y}}_{i^{\prime }}$, and ${ℐ}_{i}(l)=\{j\in ℳ(l):i\in {𝒫}_{j}\}$. The full conditional of $\omega_{i}$ is truncated normal, where truncation depends on the ith latent disease status ${\tilde{Y}}_{i}$, specifically,

$$\begin{array}{l} \omega_{i}|{\tilde{Y}}_{i},T,M∼\left\{\begin{array}{ll} TN\{\eta_{i},1,(0,\infty )\},\; & \;\text{if}\;{\tilde{Y}}_{i}=1\\ TN\{\eta_{i},1,(-\infty ,0)\},\; & \;\text{if}\;{\tilde{Y}}_{i}=0 \end{array}\right. \end{array}$$

for i=1,…,N. The notation $TN\{\mu ,\sigma^{2},(a,b)\}$ identifies a truncated normal distribution with mean μ, variance $\sigma^{2}$, and support over the interval (a,b). Sampling the sum‐of‐trees model parameters is possible given the latent variables and the form of the augmented likelihood in (4). We follow the Bayesian back fitting algorithm of Chipman et al. [19] to sample all parameters associated with the regression trees. Appendices B and C of the Supporting Information contain details and posteriors for the sum‐of‐trees model parameters. Finally, full conditional distributions of the assay accuracy probabilities are also beta, namely,

$$\begin{array}{ll} S_{e(l)}|Z,\tilde{Y} & ∼\text{beta}(a_{e(l)}^{*},b_{e(l)}^{*})\\ S_{p(l)}|Z,\tilde{Y} & ∼\text{beta}(a_{p(l)}^{*},b_{p(l)}^{*}) \end{array}$$

for l=1,…,L, where $a_{e(l)}^{*}=a_{e(l)}+\sum_{j\in ℳ(l)}Z_{j}{\tilde{Z}}_{j}$, $b_{e(l)}^{*}=b_{e(l)}+\sum_{j\in ℳ(l)}(1-Z_{j}){\tilde{Z}}_{j}$, $a_{p(l)}^{*}=a_{p(l)}+\sum_{j\in ℳ(l)}(1-Z_{j})(1-{\tilde{Z}}_{j})$, and $b_{p(l)}^{*}=b_{p(l)}+\sum_{j\in ℳ(l)}Z_{j}(1-{\tilde{Z}}_{j})$.

### 3.3 Variable Importance

After a suitable burn‐in period, our posterior algorithm returns S posterior samples of the K regression tree structures. A byproduct of BART is its ability to provide a measure of variable importance, following the approach of Chipman et al. [19]. Within a posterior sample, we can compute the proportion of times a particular covariate is used as a splitting variable among all decision rules in the ensemble of K trees. We can then estimate the variable inclusion proportion for this covariate as the mean of these proportions across the S posterior samples. In particular, let $z_{qs}$ be the number of decision rules that use the qth covariate as the splitting variable in the sth posterior draw of the sum‐of‐trees model and let $z_{.s}=\sum_{q=1}^{Q}z_{qs}$ be the total number of decision rules in the sth posterior sample. We define

$$\begin{array}{l} v_{q}=\frac{1}{S}\overset{S}{\underset{s=1}{\sum }}\frac{z_{qs}}{z_{.s}} \end{array}$$

to be the variable inclusion proportion for the qth covariate. Intuitively, covariates with large proportions $v_{q}$ are identified as influential predictors of disease status. Therefore, covariates can be ranked on the basis of their relative importance in terms of prediction. This strategy is more effective when the number of trees K is small because predictors will be forced to compete with each other to improve the fit [19]. Although this measure is widely used, it does have limitations resulting from a tendency of BART to overfit noise [26]. Thus, caution is warranted when using inclusion proportions to select a subset of important predictors.

## 4 Simulation Evidence

### 4.1 Simulation Description

This section summarizes numerical studies that evaluate the performance of BART for group testing data. We considered three population‐level models, all of which follow the form of (1), with $x_{i}={\left(x_{i1},x_{i2},x_{i3}\right)}^{\prime }$ as a vector of Q=3 covariates, $x_{i1}$ and $x_{i2}$ are uniform (0,10), and $x_{i3}∼\text{Bernoulli}(0.5)$. The true models are

$$\begin{array}{ll} \text{M1} & :\;f(x_{i})=\sin(\pi x_{i1})-1.25\\ \text{M2} & :\;f(x_{i})=\beta_{0}+\beta_{1}x_{i1}+\beta_{2}x_{i2}+\beta_{3}x_{i3}\\ \text{M3} & :\;f(x_{i})=-\sin\left(\frac{x_{i1}}{3}\right)-\sqrt{x_{i1}x_{i3}}+x_{i3} \end{array}$$

where $\beta ={\left(\beta_{0},\beta_{1},\beta_{2},\beta_{3}\right)}^{\prime }={\left(-0.85,0.55,-1.25,-0.35\right)}^{\prime }$. All three models provide a population‐level prevalence similar to the data application in Section 5. The first model (M1) was chosen to evaluate BART's performance when the data exhibit a highly non‐linear pattern. The second (M2) allows one to assess the potential loss from a BART fit when a GLM is appropriate. The third (M3) allows for non‐linear interactions emulating patterns found in the motivating data. For each model, we generated N=5000 individual true disease statuses according to ${\tilde{Y}}_{i}∼\text{Bernoulli}\{\Phi (f(x_{i})\}$. We then repeated this process 500 times independently to create 500 individual‐level data sets.

Group testing outcomes were generated for two protocols: Master pool testing (MPT) and Dorfman testing [27] (DT). Under MPT, individuals are assigned to exactly one master pool which is tested but no further testing is performed regardless of the outcome. Therefore, although it is not possible to diagnose all individuals as positive or not under MPT, this protocol can still be used to estimate the relationship between disease status and covariates. On the other hand, DT is a two‐stage protocol where positive pools from the first stage (under MPT) are resolved by individually retesting all subjects in the pools. For both protocols, we randomly assigned individuals to master pools of size 4, and the testing response for the jth pool $Z_{j}$ was generated as $Z_{j}|{\tilde{Z}}_{j}∼\text{Bernoulli}\{S_{ej}{\tilde{Z}}_{j}+(1-S_{pj})\}$, where ${\tilde{Z}}_{j}=I(\sum_{i\in {𝒫}_{j}}{\tilde{Y}}_{i}>0)$. We considered two configurations for the assay accuracy probabilities:

C1. $S_{ej}=0.95$ and $S_{pj}=0.98$, assumed to be known and the same for all J pools
C2. (for DT only) $S_{e(1)}=0.95$ and $S_{p(1)}=0.98$ are assumed for master pools in the first stage; $S_{e(2)}=0.98$ and $S_{p(2)}=0.99$ are assumed for individual tests in the second stage. These four parameters are regarded to be unknown and are estimated concurrently with the population‐level model.

Our methods are evaluated under two BART configurations: One with K=20 trees and one with K=200 trees. For the model parameters associated with the regression trees, we used the default prior specifications described in Section 2. When estimating the assay accuracy probabilities (C2), we elicited independent uniform priors for $S_{e(l)}$ and $S_{p(l)}$, for l=1,2. This represents the most challenging situation for estimation because no prior information regarding assay accuracy is introduced. We used our posterior sampling algorithm to draw S=2500 samples (after a burn‐in of 2 500 samples) and assessed convergence by using standard MCMC diagnostics. For comparison purposes, we also simultaneously estimated the Bayesian GLM from McMahan et al. [16], entering the three covariates as linear terms and specifying diffuse priors for the regression coefficients. This allows us to document the potential advantages and disadvantages of BART for group testing, noting the GLM is correctly specified for model M2 and misspecified otherwise.

All estimation results are averaged over the 500 data sets. To evaluate in‐ and out‐of‐sample classification accuracy, we performed a receiver operating characteristic (ROC) curve analysis, summarized by the area under the curve (AUC). To assess out‐of‐sample accuracy for each model fit, we simulated 1 000 new individual disease statuses using the process outlined above and then used our models to predict their disease probabilities and compute the associated AUC scores. Variable inclusion proportions $v_{q}$ were recorded for each covariate based on the S=2500 MCMC samples. For comparison, individual testing (IT) was also implemented for the configurations that assume assay accuracy probabilities $S_{ej}$ and $S_{pj}$ are known. This comparison is always of interest in group testing as it allows one to assess the information lost from pooling.

### 4.2 Simulation Results

**FIGURE 2:**
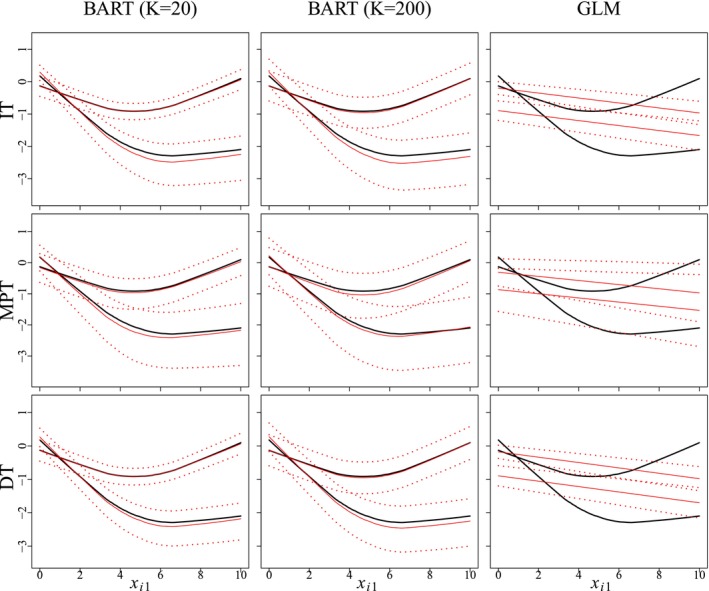
Averaged estimates of f(·) for model M3 under individual testing (IT), master pool testing (MPT), and Dorfman testing (DT). The true curve is shown in black. The solid red curve depicts the averaged posterior mean estimate based on 500 Monte Carlo data sets. The dotted curves depict 0.025 and 0.975 quantiles of the posterior mean estimates. Results for BART (with K=20 and K=200 trees) and the GLM fit [16] are shown. The same figure for model M1 is given in Appendix D of the Supporting Information.

Figure 2 summarizes the averaged estimates of f(·) under the third population‐level model (M3) for IT, MPT, and DT when assay accuracy probabilities are known. This model includes the binary covariate $x_{i3}$ so the true function, shown in solid black in Figure 2, is bifurcated depending on its value. Figure D.2, shown in Appendix D of the Supporting Information, provides the same summary for the first population‐level model (M1). In both figures, one notes the results for BART are similar when comparing the number of trees, K=20 and K=200, for both MPT and DT. Figure 2 shows that estimated functions from both BART configurations are in agreement with the true regression function for both group testing protocols under model M3. Estimation is inherently more difficult under model M1 as $f(x_{i})=\sin(\pi x_{i1})-1.25$ is a highly non‐linear function. In fact, both the MPT and DT protocols struggle to recover the “peaks and valleys” of this function exactly (see Figure D.2), but DT does produce results similar to IT. This is noteworthy as IT uses 5 000 tests, one for each individual, while DT uses a small fraction of this number. Both Figure 2 and Figure D.2 show how disparaging the fit can be when one estimates a first‐order GLM.

Table D.2 in Appendix D of the Supporting Information summarizes our ROC analyses for the in‐ and out‐of‐sample classification accuracy under all population‐level models when the assay accuracy probabilities are known. For both group testing protocols, BART has substantially better classification accuracy for models M1 and M3 than the incorrectly specified GLM. At the same time, BART performs just as well as the correctly specified GLM for model M2, showing that little is lost, at least in terms of classification accuracy, when estimating the more complex BART model with group testing data. AUC scores are consistently higher for DT when compared to MPT, and the choice of K=20 or K=200 trees does not produce noticeably different results. Figure D.4 in Appendix D of the Supporting Information plots variable inclusion proportions for both BART configurations when the assay accuracy probabilities are known. The only predictor used in model M1 is $x_{i1}$ and BART successfully identifies it as having the largest proportion among $v_{1}$, $v_{2}$, and $v_{3}$ for both group testing protocols, more noticeably when using K=20 trees. Similarly, the only predictor not used in model M3 is $x_{i2}$, and $v_{2}$ is consistently the lowest among the three proportions when this smaller number of trees is used.

Our simulation studies show the main findings summarized above remain unchanged when the assay accuracy probabilities $S_{e(1)}$, $S_{p(1)}$, $S_{e(2)}$, and $S_{p(2)}$ are unknown and are estimated alongside the population‐level models (see Configuration C2 in Section 4.1). This is significant because we used uniform distributions as prior models for these four probabilities. When these priors are used, there is simply not enough information to learn about assay accuracy under IT or MPT [16]. However, using DT affords one the opportunity to learn about these probabilities when confirmatory or counterfactual test results are observed in the two stages, for example, when a master pool tests positively in the first stage and one or more individuals in the pool test negatively in the second. In fact, Table D.1 in Appendix D of the Supporting Information shows all four probabilities are estimated correctly on average under DT for both BART configurations under all population‐level models. Furthermore, estimated coverage probabilities of nominal 95% equal‐tail credible intervals for these parameters are either within the margin of Monte Carlo error (±0.03) or slightly conservative except when one estimates a GLM incorrectly. Figures D.1 and D.3 show that averaged estimates of f(·) are largely unchanged when assay accuracy probabilities are estimated under models M1 and M3. Additional results in Appendix D convey the same findings for classification accuracy and variable inclusion.

## 5 IOWA Chlamydia Data

### 5.1 Data Description and Model

The State Hygienic Laboratory (SHL) at the University of Iowa is the largest public health laboratory in Iowa. Each year, the lab tests thousands of Iowa residents for chlamydia as part of sexually transmitted disease (STD) prevention programs. Individual endocervical swabs and urine specimens are collected from various clinics throughout the state which are then transported to the SHL. The current SHL screening procedure requires all urine specimens to be tested individually, while Dorfman testing (DT) is used for swab specimens. That is, swab specimens are first tested in master pools, usually of size 4, and positive pools are resolved by testing all specimens separately. The SHL uses the Aptima Combo 2 Assay (AC2A) to test all specimens, both pooled and individual. Pilot data describing the accuracy of the AC2A for individual testing are summarized in the assay's product literature and Gaydos et al. [28] We reproduce a portion of these data in Appendix E of the Supporting Information.

We analyze data for N=13862 female subjects tested at the SHL during the 2014 calendar year. These data consist of chlamydia test responses from 4 316 individual urine specimens, 416 individual swab specimens, 2 273 swab master pools of size 4, 12 swab master pools of size 3, one swab master pool of size 2, and additional retesting responses required to resolve positive swab master pools. We consider six covariates as potential risk factors: Age (in years, denoted by $x_{i1}$), a race indicator for the ith individual ($x_{i2}=1$ if Caucasian), an indicator denoting whether the individual reported a new sexual partner in the last 90 days ($x_{i3}=1$), an indicator denoting whether the individual reported having multiple sexual partners in the last 90 days ($x_{i4}=1$), an indicator denoting whether the individual reported sexual contact with an STD‐positive partner in the previous year ($x_{i5}=1$), and an indicator denoting whether the individual presented symptoms of infection ($x_{i6}=1$). All binary covariates were recorded as “0” if the corresponding condition was not met. To relate an individual's true chlamydia status to their available covariate information, we estimate

$$\begin{array}{l} \Phi^{-1}\{P({\tilde{Y}}_{i}=1\;|\;x_{i})\}=\overset{K}{\underset{k=1}{\sum }}g(x_{i};T_{k},M_{k}) \end{array}$$

where $x_{i}={\left(x_{i1},\ldots ,x_{i6}\right)}^{\prime }$, for i=1,…,13862. We use two BART configurations, one with K=20 trees and one with K=200 trees. We also estimated a GLM from McMahan et al. [16] for comparison, entering the six covariates in a linear fashion and eliciting non‐informative priors for the regression coefficients.

Prior distributions for the parameters in the sum‐of‐trees model and the assay accuracy probabilities described in Section 2 were used in our analysis. Although lab technicians at the SHL used the AC2A to test all specimen types, it is important to acknowledge differences in how this assay might perform when testing swab and urine specimens [28] and when testing individuals and pools. With this in mind, we divide all test responses into L=3 strata with the following AC2A accuracy probabilities: $S_{e(1)}$ and $S_{p(1)}$ for swab specimens tested individually, $S_{e(2)}$ and $S_{p(2)}$ for urine specimens tested individually, and $S_{e(3)}$ and $S_{p(3)}$ for swab specimens tested in pools. For beta prior elicitation, we first modeled these six parameters informatively using the AC2A pilot data in Appendix E of the Supporting Information. We performed separate analyses where these probabilities were modeled non‐informatively with uniform distributions over (0,1).

### 5.2 Analysis and Results

We first evaluate the predictive performance of BART on the basis of the number trees K and the prior models for the six AC2A accuracy probabilities. To do this, we randomly split the data into training and test sets, where 85% of the data was used to train the model and the remaining 15% was allocated to the test set. The true chlamydia statuses are not observed in this application due to the potential of imperfect testing. Therefore, it is not appropriate to perform a ROC curve analysis as we did in Section 4 where individual true disease statuses were simulated directly. We instead assess predictive performance through the log‐likelihood, which is analogous to assessing model‐based performance using the cross‐entropy loss function [29]. Using posterior mean parameter estimates, Table 2 reports the log‐likelihood values for the in‐ and out‐of‐sample sets under four BART configurations which arise from cross‐classifying the number of trees (K=20, K=200) and the assay prior modeling assumptions (informative, non‐informative). The BART results are nearly identical under each configuration for in‐ and out‐of‐sample data sets. Furthermore, the predictive performance of BART is superior to the GLM with linear terms only, more noticeably when informative priors for the AC2A probabilities are used.

**TABLE 2:** Iowa chlamydia data. In‐ and out‐of‐sample log‐likelihood calculated using posterior mean estimates. Results are shown for informative and non‐informative priors for the AC2A accuracy probabilities. Results from the GLM fit [16].

AC2A priors		BART (K=20)	BART (K=200)	GLM
Informative	In‐Sample	−3329.85	−3320.62	−4379.05
	Out‐of‐Sample	−595.75	−594.95	−802.07
Non‐informative	In‐Sample	−3334.01	−3326.15	−3446.61
	Out‐of‐Sample	−595.98	−597.44	−616.18

**FIGURE 3:**
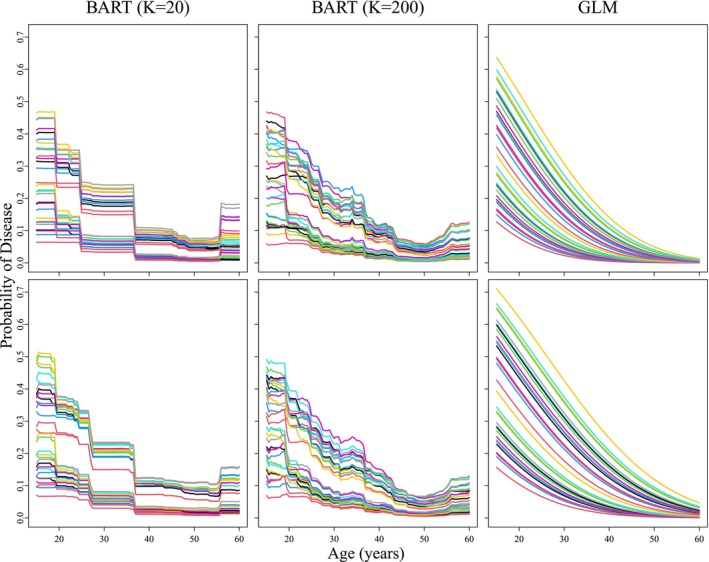
Posterior estimates of the probability of chlamydial disease as a function of age for 32 cohorts. Top row: Informative priors for AC2A accuracy probabilities. Bottom row: Non‐informative priors. BART results are shown for K=20 and K=200 trees. Results from the GLM fit [16] are also shown.

Figure 3 shows estimated probabilities of chlamydial disease as a function of age for $2^{5}=32$ risk profiles, created by forming all possible combinations of the five binary covariates (defined in Section 5.1). These estimated profiles are shown under both informative prior assumptions for the AC2A probabilities (top row) and non‐informative priors (bottom row), and we include the first‐order GLM results for comparison. The same non‐linear age effect is seen for both choices of K under BART, but the K=200 configuration provides smoother functional estimates as expected. Furthermore, estimated risk profiles that cross suggest the presence of interaction among the binary covariates and age. BART flexibly captures such interactions without having to specify the functional form of the relationship between disease status and covariates. It is interesting that BART detects a possible increase in chlamydial risk after the age of 50 years; however, it may be an overreaction to infer this for the population of Iowa females. Our data set included only 82 females aged 50 years or older (out of 13 862 total), and most of these subjects visited clinics primarily for STD services.

**FIGURE 4:**
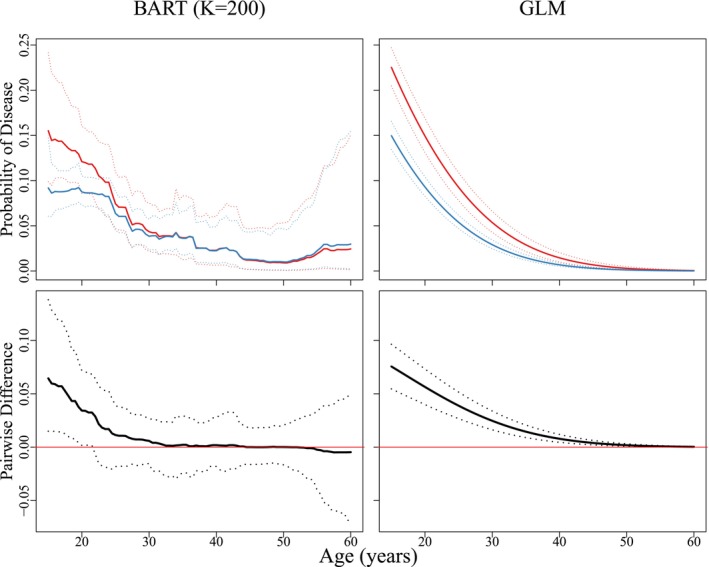
Top: The solid red curve denotes posterior estimates of the probability of chlamydial disease for non‐Caucasian females who reported having a new sexual partner ($x_{i2}=0$, $x_{i3}=1$). The solid blue curve denotes the same estimates for Caucasian females ($x_{i2}=1$, $x_{i3}=1$). Bottom: Difference in the estimated probabilities for the two cohorts in the top row. Pointwise 95% equal‐tail credible bands are shown dotted. These figures were constructed by assuming informative priors for the AC2A accuracy probabilities and K=200 trees. The same figure under non‐informative priors is shown in Web Appendix F of the Supporting Information.

To further highlight the flexibility of BART when analyzing group testing data, Figure 4 (top row) shows estimated probabilities of disease as a function of age for two cohorts of subjects: 582 non‐Caucasian females who reported having a new sexual partner ($x_{i2}=0$, $x_{i3}=1$) and 3 199 Caucasian females who reported having a new sexual partner ($x_{i2}=1$, $x_{i3}=1$). The bottom row of Figure 4 plots the difference in the estimates for these two cohorts. The corresponding estimates from fitting a first‐order GLM are also shown for comparison purposes, and all subfigures include pointwise 95% equal‐tail credible bands. The two cohorts identified in Figure 4 are potentially of interest from a screening policy point of view. The United States Preventive Services Task Force [30] has recommended annual chlamydia testing for all sexually active females in the United States aged 24 years and younger. Our BART analysis produces results that are supportive of this recommendation and furthermore detects a significant difference between younger females in these two groups. Specifically, the probability of disease is significantly higher for non‐Caucasian females up until the age of approximately 22 years but is not statistically different after this age. Finally, note that Figure 4 was constructed using our BART model estimates with K=200 trees under informative prior distributions for the AC2A accuracy probabilities. The same figure under non‐informative priors reveals similar findings and is shown in Appendix F of the Supporting Information. This appendix also includes additional Iowa data analysis results, including variable importance proportions and estimates of the AC2A accuracy probabilities under both informative and non‐informative prior model assumptions.

## 6 Discussion

We have developed a general BART regression framework for group testing data with individual‐level covariates. Our methods expand on the Bayesian approach in McMahan et al. [16] to seamlessly incorporate non‐linear main effects and potentially high‐order interaction effects while estimating assay accuracy probabilities. BART can also assess variable importance by examining relative frequencies with which covariates are used as splitting variables in the posterior samples. R code for data analysis is available on the first author's GitHub website https://github.com/mstville/bart_gt.

In our survey of the group testing literature for regression analysis, we can find no other approach as flexible as the one presented in this paper. At the same time, several modeling enhancements could be of interest, including BART and other machine learning methods for group testing data with random effects, [11, 17] pool responses which are subject to dilution [31, 32], and missing covariates [12]. Another research direction would be to develop flexible regression methods for multivariate binary outcomes arising from group testing with multiplex assays, that is, assays that test pooled specimens for multiple diseases at once. Group testing case identification protocols with multiplex assays have been proposed in Tebbs et al. [33], Hou et al. [34], and Hou et al. [35].

## Conflicts of Interest

The authors declare no conflicts of interest.

## Supporting information

Supporting Information.

## Data Availability

The data that support the findings of this study are available from the corresponding author upon reasonable request.
