# Supplementary material for: Bayesian Additive Regression Trees for Group Testing Data
Source: Stat Med. 2025 Mar 14;44(6):e70052. doi: 10.1002/sim.70052 (PMC11907685; doi:10.1002/sim.70052)
Supplement: Supplementary file 1 — Supporting Information. [file SIM-44-0-s001.pdf]

Supporting Information for "Bayesian Additive Regression Trees for Group Testing Data"

Madeleine E. St. Ville[1], Christopher S. McMahan[2], Joe D. Bible[2], Joshua M. Tebbs[3], and Christopher R. Bilder[4]

[1]*Eunice Kennedy Shriver* National Institute of Child Health and Human Development, National Institutes of Health, Bethesda, MD, U.S.A

[2]School of Mathematical and Statistical Sciences, Clemson University, Clemson, SC, U.S.A.

[3]Department of Statistics, University of South Carolina, Columbia, SC, U.S.A.

[4]Department of Statistics, University of Nebraska-Lincoln, Lincoln, NE, U.S.A.

Appendix A. *Posterior sampling algorithm.* The complete posterior algorithm to estimate our BART regression model is given below. All notation is defined in the manuscript.

1. Initialize $(T_1^{(0)}, M_1^{(0)}), \ldots, (T_K^{(0)}, M_K^{(0)})$ and $\widetilde{Y}_i^{(0)}$ for $i = 1, \ldots, N$. If estimating the assay accuracy probabilities, initialize $\boldsymbol{S}_e^{(0)}$ and $\boldsymbol{S}_p^{(0)}$. Otherwise, set $\boldsymbol{S}_e^{(s)} = \boldsymbol{S}_e$ and $\boldsymbol{S}_p^{(s)} = \boldsymbol{S}_p$ for all s. Set $s = 1$.

2. For $i = 1, \ldots, N$, sample
$$\omega_i^{(s)} \sim \begin{cases} TN\left[\eta_i, 1, (0, \infty)\right], & \text{if } \widetilde{Y}_i^{(s-1)} = 1 \\ TN\left[\eta_i, 1, (-\infty, 0)\right], & \text{if } \widetilde{Y}_i^{(s-1)} = 0, \end{cases}$$
where $\eta_i = \eta(\boldsymbol{x}_i) = \sum_{k=1}^{K} g(\boldsymbol{x}_i; T_k^{(s-1)}, M_k^{(s-1)})$. Aggregate $\boldsymbol{\omega}^{(s)} = (\omega_1^{(s)}, \ldots, \omega_N^{(s)})'$.

3. For $k = 1, \ldots, K$, sample $(T_k^{(s)}, M_k^{(s)})$ from $\pi((T_k, M_k) \,\big|\, (\boldsymbol{T}_{-k}^{(s)}, \boldsymbol{M}_{-k}^{(s)}), \boldsymbol{\omega}^{(s)})$. The collection $(\boldsymbol{T}_{-k}^{(s)}, \boldsymbol{M}_{-k}^{(s)})$ is $(T_1^{(s)}, M_1^{(s)}), \ldots, (T_K^{(s)}, M_K^{(s)})$ with $(T_k^{(s)}, M_k^{(s)})$ removed; see Appendix B. Obtain $\eta_i = \sum_{k=1}^{K} g(\boldsymbol{x}_i; T_k^{(s)}, M_k^{(s)})$ for $i = 1, \ldots, N$.

4. If estimating the assay accuracy probabilities, sample $S_{e(l)}^{(s)} \sim \text{beta}(a_{e(l)}^*, b_{e(l)}^*)$ and $S_{p(l)}^{(s)} \sim \text{beta}(a_{p(l)}^*, b_{p(l)}^*)$, for $l = 1, ..., L$, where $a_{e(l)}^*$, $b_{e(l)}^*$, $a_{p(l)}^*$, and $b_{p(l)}^*$ are evaluated at $\widetilde{\boldsymbol{Y}}^{(s-1)}$. Aggregate $\boldsymbol{S}_e^{(s)} = (S_{e(1)}^{(s)}, \ldots, S_{e(L)}^{(s)})'$ and $\boldsymbol{S}_p^{(s)} = (S_{p(1)}^{(s)}, \ldots, S_{p(L)}^{(s)})'$.

5. For $i = 1, \ldots, N$, sample
$$\widetilde{Y}_i^{(s)} \sim \text{Bernoulli}\left(\frac{p_{i1}^*}{p_{i0}^* + p_{i1}^*}\right),$$
where p_{i0}^* and p_{i1}^* are evaluated at $\widetilde{\boldsymbol{Y}}_{-i}^{(s)} = (\widetilde{Y}_1^{(s)}, \ldots, \widetilde{Y}_{i-1}^{(s)}, \widetilde{Y}_{i+1}^{(s-1)}, \ldots, \widetilde{Y}_N^{(s-1)})'$, $\boldsymbol{S}_e^{(s)}$, $\boldsymbol{S}_p^{(s)}$, and $\eta_i = \sum_{k=1}^{K} g(\boldsymbol{x}_i; T_k^{(s)}, M_k^{(s)})$.

6. Increment $s = s + 1$ and return to Step 2.

Appendix B. *BART backfitting algorithm details.* We describe the Bayesian backfitting MCMC algorithm, outlined in Chipman et al. (2010), to sample from the posterior distribution of the regression trees. The algorithm is a Gibbs sampler that employs a modified version of Bayesian backfitting MCMC introduced by Hastie and Tibshirani (2000). An iteration of the algorithm first requires N successive draws of the latent random variables ω_i that were introduced in the second stage of our data augmentation procedure, that is,

$$\omega_i \sim \begin{cases} TN\{\eta_i, 1, (0, \infty)\}, & \text{if } \widetilde{Y}_i = 1 \\ TN\{\eta_i, 1, (-\infty, 0)\}, & \text{if } \widetilde{Y}_i = 0. \end{cases}$$

We then treat the latent variables ω_i as continuous outcomes and recast our BART model as

$$\omega_i = \eta(\boldsymbol{x}_i) + \epsilon_i, \tag{B.1}$$

for $i = 1, \ldots, N$, where $\epsilon_i \overset{\text{iid}}{\sim} N(0, 1)$. Following this, the algorithm then requires K successive draws of the individual trees (T_k, M_k) conditioning on the remaining $K - 1$ trees; i.e.,

$$\pi((T_k, M_k) \bigm| \boldsymbol{T}_{-k}, \boldsymbol{M}_{-k}, \boldsymbol{\omega})), \tag{B.2}$$

where $\boldsymbol{T}_{-k}$ is the set of $K-1$ tree structures excluding T_k and $\boldsymbol{M}_{-k}$ are the associated terminal node parameters. To obtain a draw from (B.2), note that $\pi((T_k, M_k) \bigm| \boldsymbol{T}_{-k}, \boldsymbol{M}_{-k}, \boldsymbol{\omega}))$ depends on $(\boldsymbol{T}_{-k},\ \boldsymbol{M}_{-k},\ \boldsymbol{\omega})$ through the kth vector set of partial residuals $\boldsymbol{R}_k = (R_{k1}, \ldots, R_{kN})'$, where the ith element of $\boldsymbol{R}_k$ is

$$R_{ki} = \omega_i - \sum_{u \neq k}^{K} g(\boldsymbol{x}_i; T_u, M_u),$$

for $i = 1, \ldots, N$. Thus, the model (B.1) can be temporarily reparameterized in terms of these partial residuals, that is, $R_{ki} \sim N(g(\boldsymbol{x}_i; T_k, M_k), 1)$, and a posterior draw from (B.2) is equivalent to a posterior draw from $\pi((T_k, M_k) \bigm| \boldsymbol{R}_k)$. We can obtain a draw from $\pi((T_k, M_k) \bigm| \boldsymbol{R}_k)$ in two steps. Because a conjugate normal prior on μ_{kt} is used, for $t = 1, \ldots, b_k$, we can first integrate out M_k and sample from $\pi(T_k \mid \boldsymbol{R}_k)$. We can then draw from $\pi(M_k \mid T_k, \boldsymbol{R}_k)$. We obtain a draw from $\pi(T_k \mid \boldsymbol{R}_k)$ by using the Metropolis-Hastings algorithm of Chipman et al. (1998), where we first generate a candidate tree T_k^* with probability distribution $q(T_k, T_k^*)$ and accept T_k^* with probability

$$\alpha(T_k, T_k^*) = \min\left\{1,\ \frac{q(T_k^*, T_k)}{q(T_k, T_k^*)} \frac{p(\boldsymbol{R}_k \mid T_k^*, M_k)}{p(\boldsymbol{R}_k \mid T_k, M_k)} \frac{\pi(T_k^*)}{\pi(T_k)}\right\},$$

where $q(T_k^*, T_k)/q(T_k, T_k^*)$ is the transition ratio, $p(\boldsymbol{R}_k \mid T_k^*, M_k)/p(\boldsymbol{R}_k \mid T_k, M_k)$ is the likelihood ratio, and $\pi(T_k^*)/\pi(T_k)$ is the tree structure ratio. A new tree T_k^* can be proposed given the current tree T_k using one of four moves: growing a terminal node, pruning a pair of terminal nodes, swapping the splitting criteria of two non-terminal nodes, and changing the splitting criteria of a non-terminal node; see Chipman et al. (1998, 2010). Once we have the draw from $\pi(T_k \mid \boldsymbol{R}_k)$, the posterior draw from $\pi(M_k \mid T_k, \boldsymbol{R}_k)$ is a set of independent draws of the terminal node parameters μ_{kt} from a normal distribution; see Appendix C.

Appendix C. *Posterior distribution of* μ_{kt}. Let $\boldsymbol{R}_{k(t)} = (R_{k(t)1}, ..., R_{k(t)n_t})'$ be the n_t-dimensional subset vector of $\boldsymbol{R}_k$, where n_t is the number of elements of $\boldsymbol{R}_k$ allocated to the terminal node parameter μ_{kt}. Note that $R_{k(t)h} \mid T_k, M_k \sim N(\mu_{kt}, 1)$, for $h = 1, \ldots, n_t$, and $\mu_{kt} \mid T_k \sim N(0, \sigma_\mu^2)$. Therefore,

$$\begin{aligned}
\pi(\mu_{kt} \mid T_k, \boldsymbol{R}_k) &\propto \pi(\boldsymbol{R}_{k(t)} \mid T_k, \mu_{kt})\pi(\mu_{kt} \mid T_k) \\
&\propto \exp\left\{-\frac{\sum_h (R_{k(t)h} - \mu_{kt})^2}{2}\right\} \exp\left(-\frac{\mu_{kt}^2}{2\sigma_\mu^2}\right) \\
&\propto \exp\left\{-\frac{(n_t\sigma_\mu^2 + 1)\mu_{kt}^2 - 2(\sigma_\mu^2 \sum_h R_{k(t)h})\mu_{kt}}{2\sigma_\mu^2}\right\} \\
&\propto \exp\left\{-\frac{\left(\mu_{kt} - \frac{\sigma_\mu^2 \sum_h R_{k(t)h}}{n_t\sigma_\mu^2+1}\right)^2}{2\left(\frac{\sigma_\mu^2}{n_t\sigma_\mu^2+1}\right)}\right\}.
\end{aligned}$$

This shows the posterior distribution

$$\mu_{kt} \mid T_k, \boldsymbol{R}_k \sim N\left(\frac{\sigma_\mu^2 \sum_h R_{k(t)h}}{n_t\sigma_\mu^2 + 1}, \frac{\sigma_\mu^2}{n_t\sigma_\mu^2 + 1}\right).$$

Appendix D. *Additional simulation results.* This appendix gives additional simulation results for our numerical studies in Section 4 in the manuscript. We consider the three population-level models:

$$\begin{aligned}
\text{M1}: &\quad f(\boldsymbol{x}_i) = \sin(\pi x_{i1}) - 1.25 \\
\text{M2}: &\quad f(\boldsymbol{x}_i) = \beta_0 + \beta_1 x_{i1} + \beta_2 x_{i2} + \beta_3 x_{i3} \\
\text{M3}: &\quad f(\boldsymbol{x}_i) = -\sin\left(\frac{x_{i1}}{3}\right) - \sqrt{x_{i1}x_{i3}} + x_{i3},
\end{aligned}$$

where $\boldsymbol{\beta} = (\beta_0, \beta_1, \beta_2, \beta_3)' = (-0.85, 0.55, -1.25, -0.35)'$. Figure 2 in the manuscript summarizes the averaged estimates of $f(\cdot)$ under M3 for individual testing (IT), master pool testing (MPT), and Dorfman testing (DT) when the assay accuracy probabilities are known. The following figures and table accompany Figure 2:

- Figure D.1. Model M3 when assay accuracy probabilities are unknown (DT only)
- Figure D.2. Model M1 when assay accuracy probabilities are known
- Figure D.3. Model M1 when assay accuracy probabilities are unknown (DT only)
- Table D.1. Assay accuracy probability estimation performance for all three population-level models (DT only).

These are shown on pages 4-7.

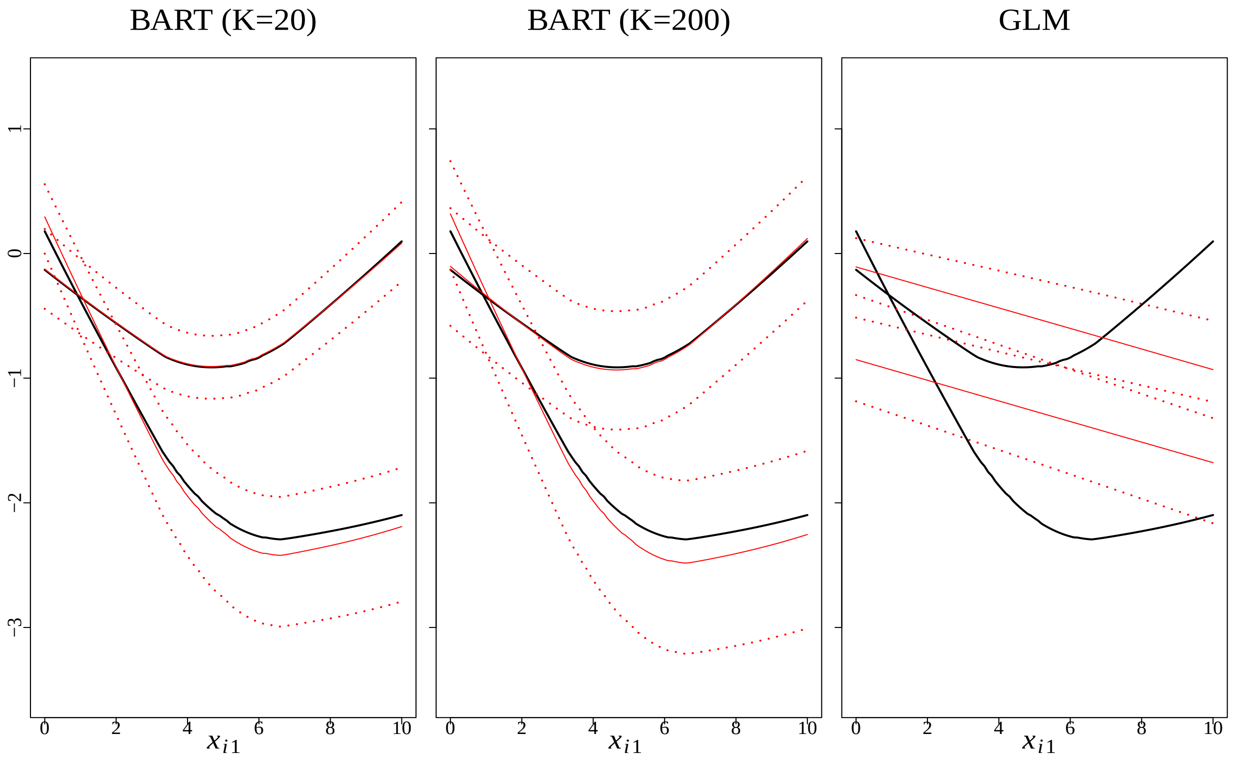

Figure D.1: Averaged estimates of $f(\cdot)$ for model M3 under Dorfman testing (DT) when assay accuracy probabilities are unknown. The true curve is shown in black. The solid red curve depicts the averaged posterior mean estimate based on 500 Monte Carlo data sets. Dotted curves depict 0.025 and 0.975 quantiles of the posterior mean estimates. Results for BART (with $K = 20$ and $K = 200$ trees) and the GLM fit (McMahan et al., 2017) are shown.

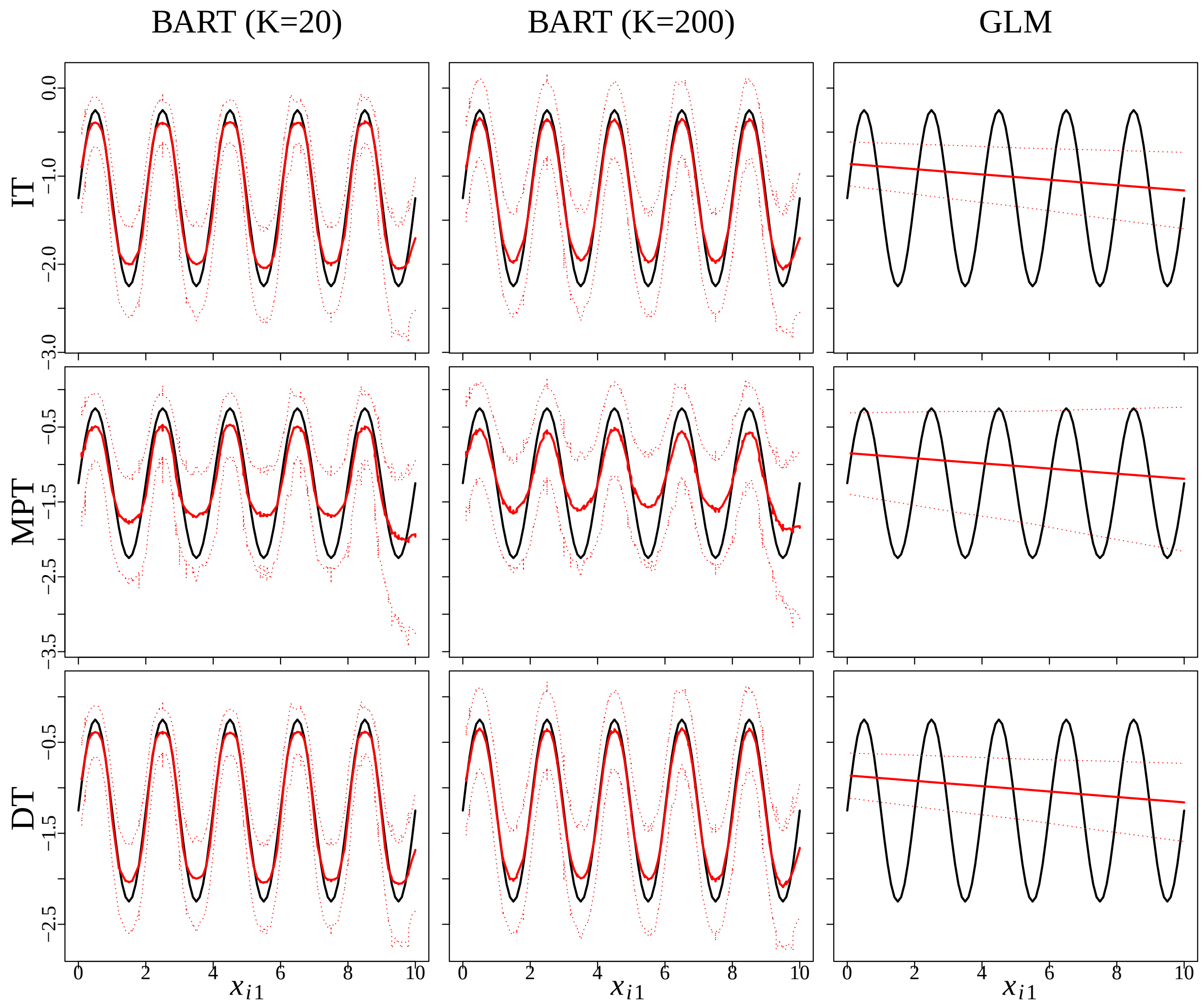

Figure D.2: Averaged estimates of $f(\cdot)$ for model M1 under individual testing (IT), master pool testing (MPT), and Dorfman testing (DT). The true curve is shown in black. The solid red curve depicts the averaged posterior mean estimate based on 500 Monte Carlo data sets. Dotted curves depict 0.025 and 0.975 quantiles of the posterior mean estimates. Results for BART (with $K = 20$ and $K = 200$ trees) and the GLM fit (McMahan et al., 2017) are shown.

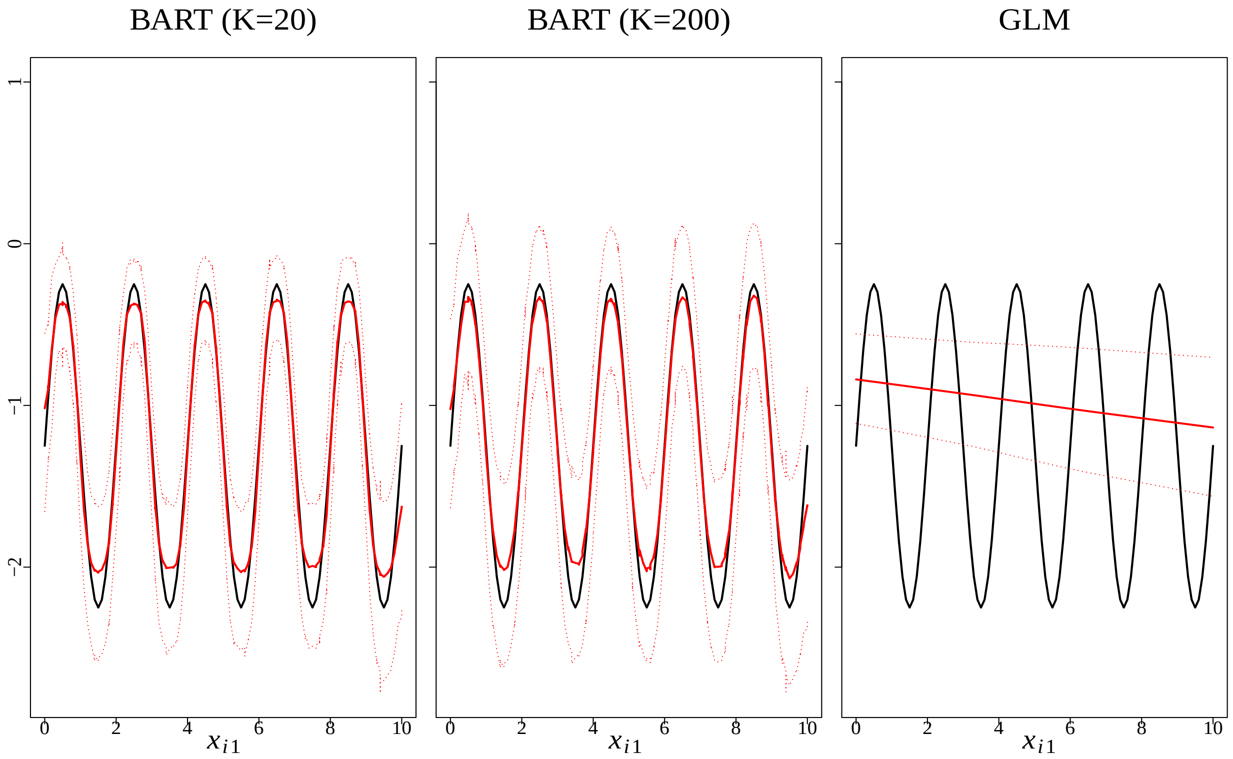

Figure D.3: Averaged estimates of $f(\cdot)$ for model M1 under Dorfman testing (DT) when assay accuracy probabilities are unknown. The true curve is shown in black. The solid red curve depicts the averaged posterior mean estimate based on 500 Monte Carlo data sets. Dotted curves depict 0.025 and 0.975 quantiles of the posterior mean estimates. Results for BART (with $K = 20$ and $K = 200$ trees) and the GLM fit (McMahan et al., 2017) are shown.

Table D.1: Unknown assay accuracy probabilities: Estimation performance under models M1, M2, and M3 for DT. Average bias and sample standard deviation (SSD) of 500 posterior mean estimates, averaged estimated posterior standard deviation (ESE) and empirical coverage probabilities of nominal 95% equal-tail credible intervals (CP95). Results for BART (with $K = 20$ and $K = 200$ trees) and the GLM fit (McMahan et al., 2017) are shown.

Model	Method		$S_{e(1)} = 0.95$	$S_{p(1)} = 0.98$	$S_{e(2)} = 0.98$	$S_{p(2)} = 0.99$
M1	BART ($K = 20$)	Bias (CP95)	-0.02 (0.97)	-0.00 (0.99)	-0.01 (1.00)	-0.00 (0.98)
		SSD (ESE)	0.03 (0.04)	0.01 (0.01)	0.01 (0.01)	0.00 (0.01)
	BART ($K = 200$)	Bias (CP95)	-0.02 (0.97)	-0.00 (0.99)	-0.00 (0.99)	0.00 (0.99)
		SSD (ESE)	0.03 (0.04)	0.01 (0.01)	0.01 (0.01)	0.00 (0.01)
	GLM	Bias (CP95)	-0.03 (1.00)	0.00 (1.00)	-0.01 (1.00)	-0.00 (1.00)
		SSD (ESE)	0.02 (0.05)	0.00 (0.01)	0.01 (0.02)	0.00 (0.01)
M2	BART ($K = 20$)	Bias (CP95)	-0.01 (0.93)	-0.00 (0.95)	-0.00 (0.93)	-0.00 (0.94)
		SSD (ESE)	0.01 (0.01)	0.01 (0.01)	0.01 (0.01)	0.00 (0.00)
	BART ($K = 200$)	Bias (CP95)	-0.01 (0.95)	-0.00 (0.93)	-0.00 (0.93)	-0.00 (0.93)
		SSD (ESE)	0.01 (0.01)	0.01 (0.01)	0.01 (0.01)	0.00 (0.00)
	GLM	Bias (CP95)	-0.00 (0.95)	-0.00 (0.97)	-0.00 (0.95)	-0.00 (0.95)
		SSD (ESE)	0.01 (0.01)	0.01 (0.01)	0.01 (0.01)	0.00 (0.00)
M3	BART ($K = 20$)	Bias (CP95)	-0.01 (0.94)	-0.00 (0.99)	-0.00 (0.99)	-0.00 (0.92)
		SSD (ESE)	0.03 (0.03)	0.01 (0.01)	0.01 (0.01)	0.01 (0.01)
	BART ($K = 200$)	Bias (CP95)	-0.02 (0.92)	-0.00 (0.99)	-0.01 (0.98)	-0.00 (0.92)
		SSD (ESE)	0.03 (0.03)	0.01 (0.01)	0.01 (0.01)	0.01 (0.01)
	GLM	Bias (CP95)	-0.05 (0.77)	0.00 (1.00)	-0.01 (0.99)	0.00 (0.99)
		SSD (ESE)	0.03 (0.04)	0.01 (0.01)	0.01 (0.01)	0.00 (0.00)

We now present the results of our ROC analyses and provide variable inclusion proportions for the three population-level models. These results are summarized in the figures and tables listed below:

- Table D.2. ROC analysis when assay accuracy probabilities are known
- Figure D.4. Variable inclusion proportions when assay accuracy probabilities are known
- Table D.3. ROC analysis when assay accuracy probabilities are unknown (DT only)
- Figure D.5. Variable inclusion proportions when assay accuracy probabilities are unknown (DT only).

These tables and figures are shown on pages 8-10.

Table D.2: Average estimated AUC and sample standard deviation (in parentheses) when assay accuracy probabilities are known.

Model	Protocol		BART ($K = 20$)	BART ($K = 200$)	GLM
M1	IT	In-Sample	0.80 (0.01)	0.81 (0.01)	0.54 (0.01)
		Out-of-Sample	0.77 (0.02)	0.78 (0.02)	0.53 (0.02)
	MPT	In-Sample	0.76 (0.01)	0.77 (0.01)	0.54 (0.01)
		Out-of-Sample	0.74 (0.02)	0.75 (0.02)	0.52 (0.02)
	DT	In-Sample	0.80 (0.01)	0.81 (0.01)	0.54 (0.01)
		Out-of-Sample	0.77 (0.02)	0.78 (0.02)	0.53 (0.02)
M2	IT	In-Sample	0.99 (0.00)	0.99 (0.00)	0.99 (0.00)
		Out-of-Sample	0.98 (0.00)	0.98 (0.00)	0.98 (0.00)
	MPT	In-Sample	0.98 (0.00)	0.99 (0.00)	0.98 (0.00)
		Out-of-Sample	0.97 (0.00)	0.98 (0.00)	0.98 (0.00)
	DT	In-Sample	0.99 (0.00)	0.99 (0.00)	0.98 (0.00)
		Out-of-Sample	0.98 (0.00)	0.98 (0.00)	0.98 (0.00)
M3	IT	In-Sample	0.82 (0.01)	0.83 (0.01)	0.69 (0.01)
		Out-of-Sample	0.80 (0.02)	0.80 (0.01)	0.70 (0.02)
	MPT	In-Sample	0.80 (0.01)	0.81 (0.01)	0.68 (0.01)
		Out-of-Sample	0.79 (0.02)	0.79 (0.02)	0.69 (0.02)
	DT	In-Sample	0.82 (0.01)	0.83 (0.01)	0.69 (0.01)
		Out-of-Sample	0.80 (0.01)	0.80 (0.02)	0.70 (0.01)

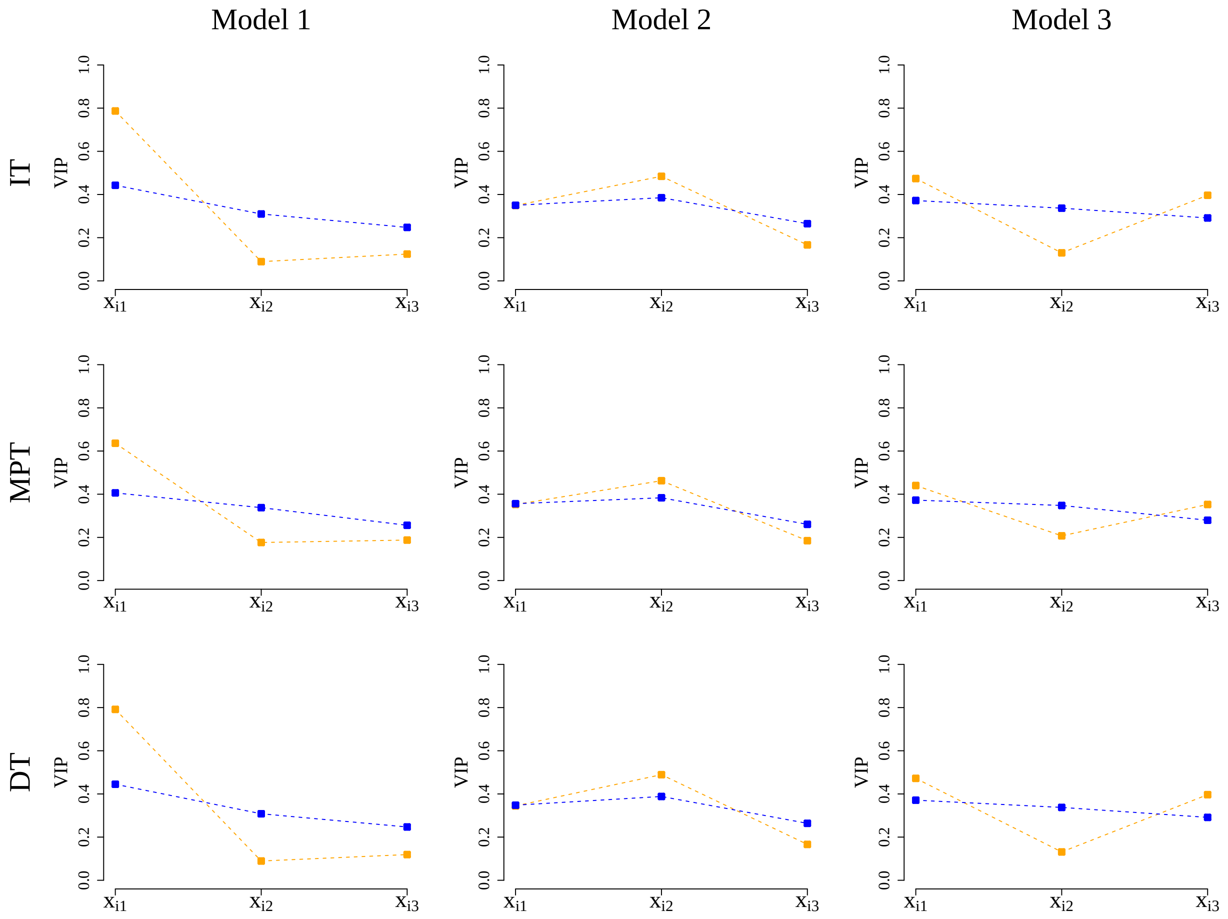

Figure D.4: Variable inclusion proportions when assay accuracy probabilities are known. Results for $K = 20$ trees are shown in orange. Results for $K = 200$ trees are shown in blue.

Table D.3: Average estimated AUC and sample standard deviation (in parentheses) when assay accuracy probabilities are unknown. Results for DT are shown.

Model		BART ($K = 20$)	BART ($K = 200$)	GLM
M1	In-Sample	0.80 (0.01)	0.82 (0.01)	0.54 (0.01)
	Out-of-Sample	0.77 (0.02)	0.78 (0.02)	0.53 (0.02)
M2	In-Sample	0.99 (0.00)	0.99 (0.00)	0.99 (0.00)
	Out-of-Sample	0.98 (0.00)	0.98 (0.00)	0.98 (0.00)
M3	In-Sample	0.82 (0.01)	0.83 (0.01)	0.69 (0.01)
	Out-of-Sample	0.80 (0.02)	0.80 (0.02)	0.70 (0.01)

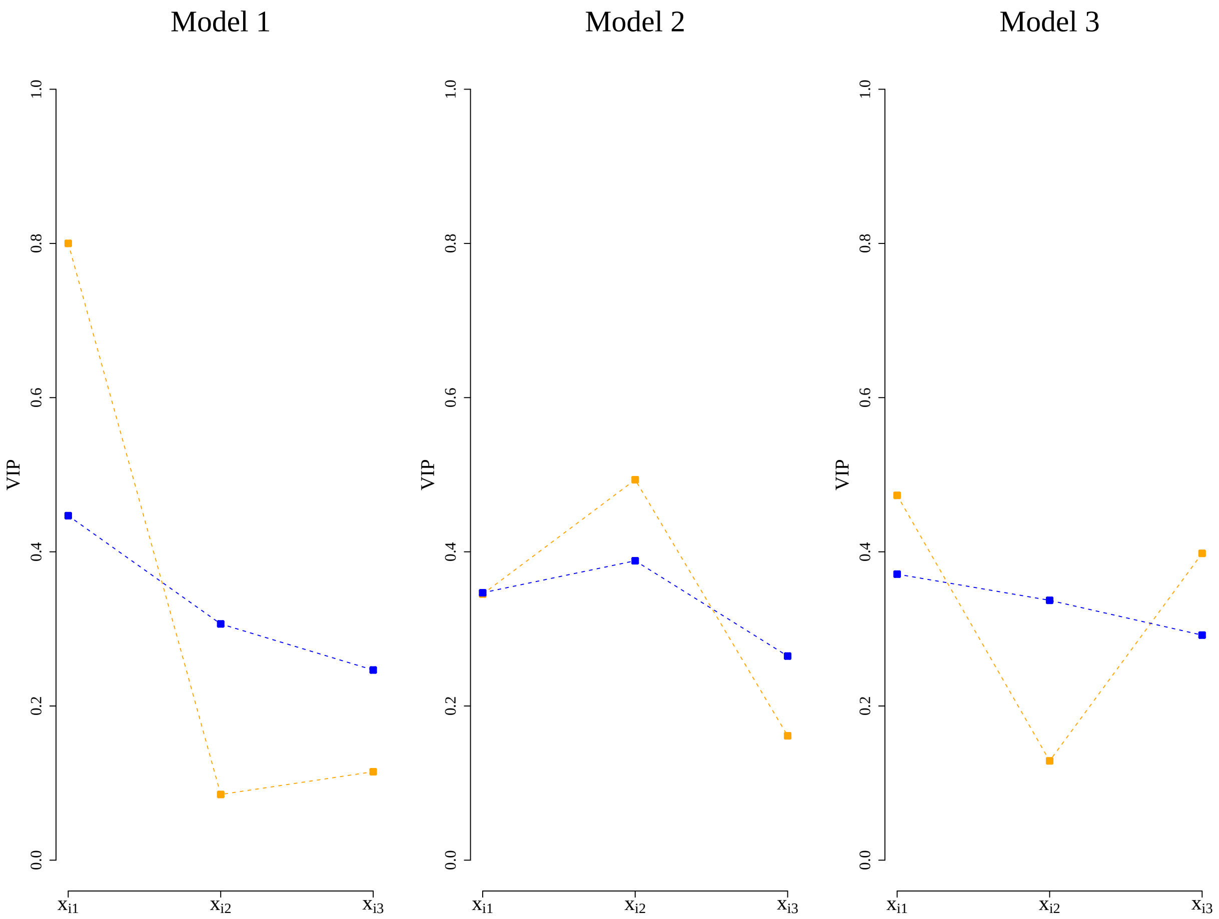

Figure D.5: Variable inclusion proportions when assay accuracy probabilities are unknown (DT only). Results for $K = 20$ trees are shown in orange. Results for $K = 200$ trees are shown in blue.

Appendix E. *AC2A pilot data.* This appendix reproduces the pilot study results published in the AC2A product literature and Gaydos et al. (2003) for female swab and urine specimens.

Table E.1: AC2A pilot data for female specimens.

Specimen type	TP	FN	TN	FP
Swab	195	12	1154	28
Urine	197	11	1170	13

The notation used in this table is defined below:

$$\begin{aligned}\text{TP} &= \text{number of true positive individual test results}\\ \text{FN} &= \text{number of false negative individual test results}\\ \text{TN} &= \text{number of true negative individual test results}\\ \text{FP} &= \text{number of false positive individual test results.}\end{aligned}$$

These results are used to set informative priors for the AC2A accuracy probabilities. Sensitivity parameters in Section 5 use beta priors with hyperparameters

$$\begin{aligned} a_{e(l)} &= \text{TP} + 1 \\ b_{e(l)} &= \text{FN} + 1. \end{aligned}$$

Specificity parameters use hyperparameters

$$\begin{aligned} a_{p(l)} &= \text{TN} + 1 \\ b_{p(l)} &= \text{FP} + 1. \end{aligned}$$

These create prior distributions concentrated around TP/(TP + FN) and TN/(TN + FP), respectively. For swab specimens, the priors are beta$(196, 13)$ and beta$(1155, 29)$, respectively. For urine specimens, the priors are beta$(198, 12)$ and beta$(1171, 14)$, respectively.

Appendix F. *Additional Iowa data results.* This appendix gives additional results for our Iowa data analysis in Section 5 in the manuscript.

The following figure and table accompany the results in Section 5 when using informative prior distributions for the AC2A accuracy probabilities:

- Figure F.1. Variable inclusion proportions
- Table F.1. AC2A accuracy estimation results.

The following figures and table accompany the results in Section 5 when using noninformative prior distributions for the AC2A accuracy probabilities:

- Figure F.2. Estimated probabilities of disease as a function of age and pairwise differences for the two cohorts in Figure 4 in the manuscript
- Figure F.3. Variable inclusion proportions
- Table F.2. AC2A accuracy estimation results.

These are shown on pages 13-18.

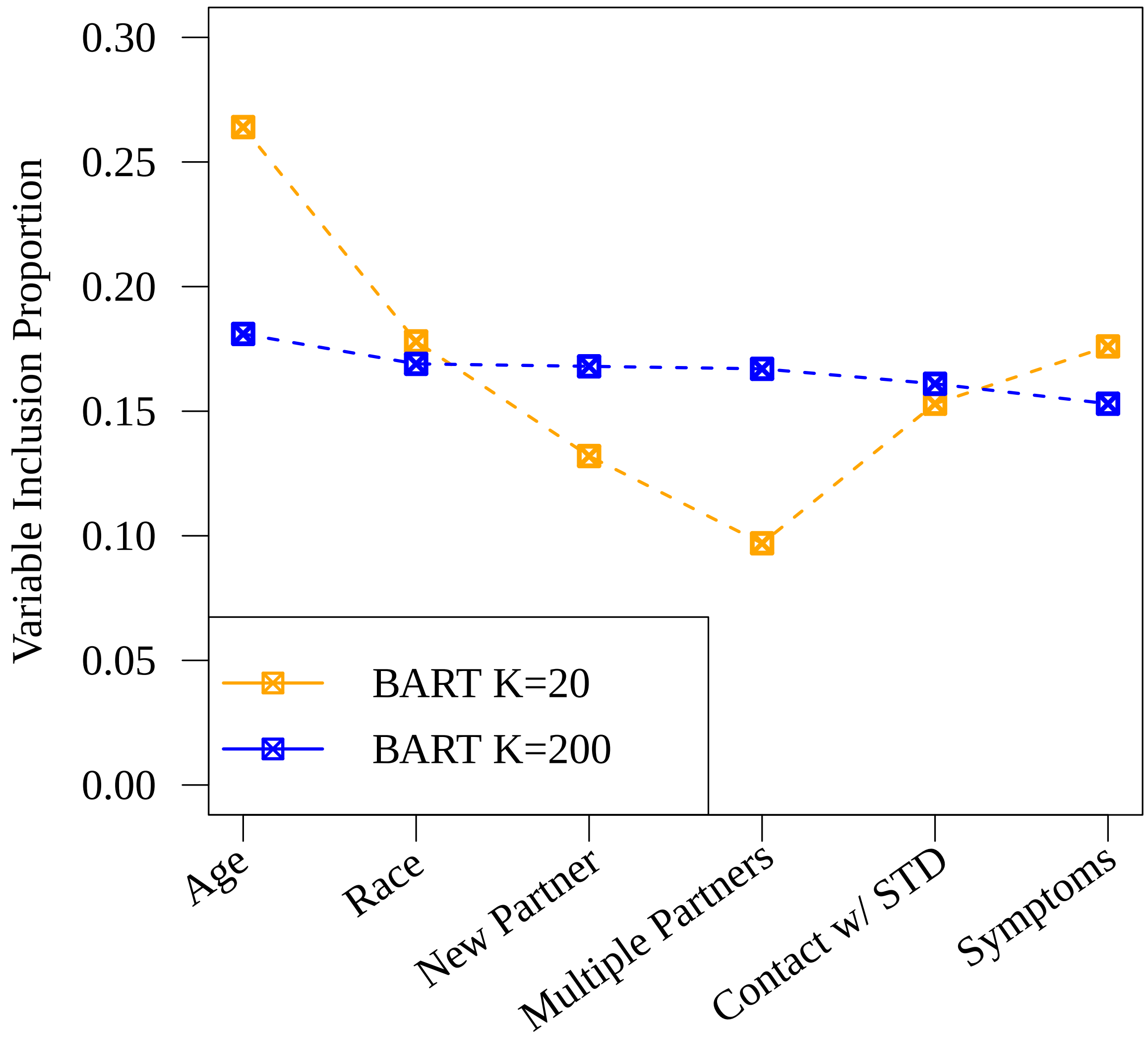

Figure F.1: Variable inclusion proportions using informative priors for the AC2A accuracy probabilities.

Table F.1: Estimation results for AC2A accuracy probabilities under informative priors. The posterior mean estimate (Est.), the estimated posterior standard deviation (ESE), and the 95% equal-tail credible interval (CI95) are shown. Results are shown for BART with $K = 20$ and $K = 200$ trees and the GLM fit (McMahan et al., 2017). All table values are shown using 2 decimal places.

		BART ($K = 20$)			BART ($K = 200$)			GLM		
		Est.	ESE	CI95	Est.	ESE	CI95	Est	ESE	CI95
$S_{e(1)}$	Swab Individual	0.98	0.00	(0.97, 0.99)	0.98	0.00	(0.97, 0.99)	0.97	0.04	(0.83, 0.99)
$S_{e(2)}$	Urine Individual	0.94	0.02	(0.91, 0.97)	0.95	0.02	(0.91, 0.97)	0.90	0.10	(0.56, 0.96)
$S_{e(3)}$	Swab Pool	0.94	0.02	(0.91, 0.97)	0.94	0.02	(0.91, 0.97)	0.90	0.10	(0.57, 0.97)
$S_{p(1)}$	Swab Individual	0.97	0.00	(0.97, 0.98)	0.97	0.00	(0.97, 0.98)	0.97	0.00	(0.96, 0.98)
$S_{p(2)}$	Urine Individual	0.99	0.00	(0.98, 0.99)	0.99	0.00	(0.98, 0.99)	0.99	0.00	(0.98, 0.99)
$S_{p(3)}$	Swab Pool	0.99	0.00	(0.99, 0.99)	0.99	0.00	(0.99, 0.99)	0.99	0.00	(0.98, 0.99)

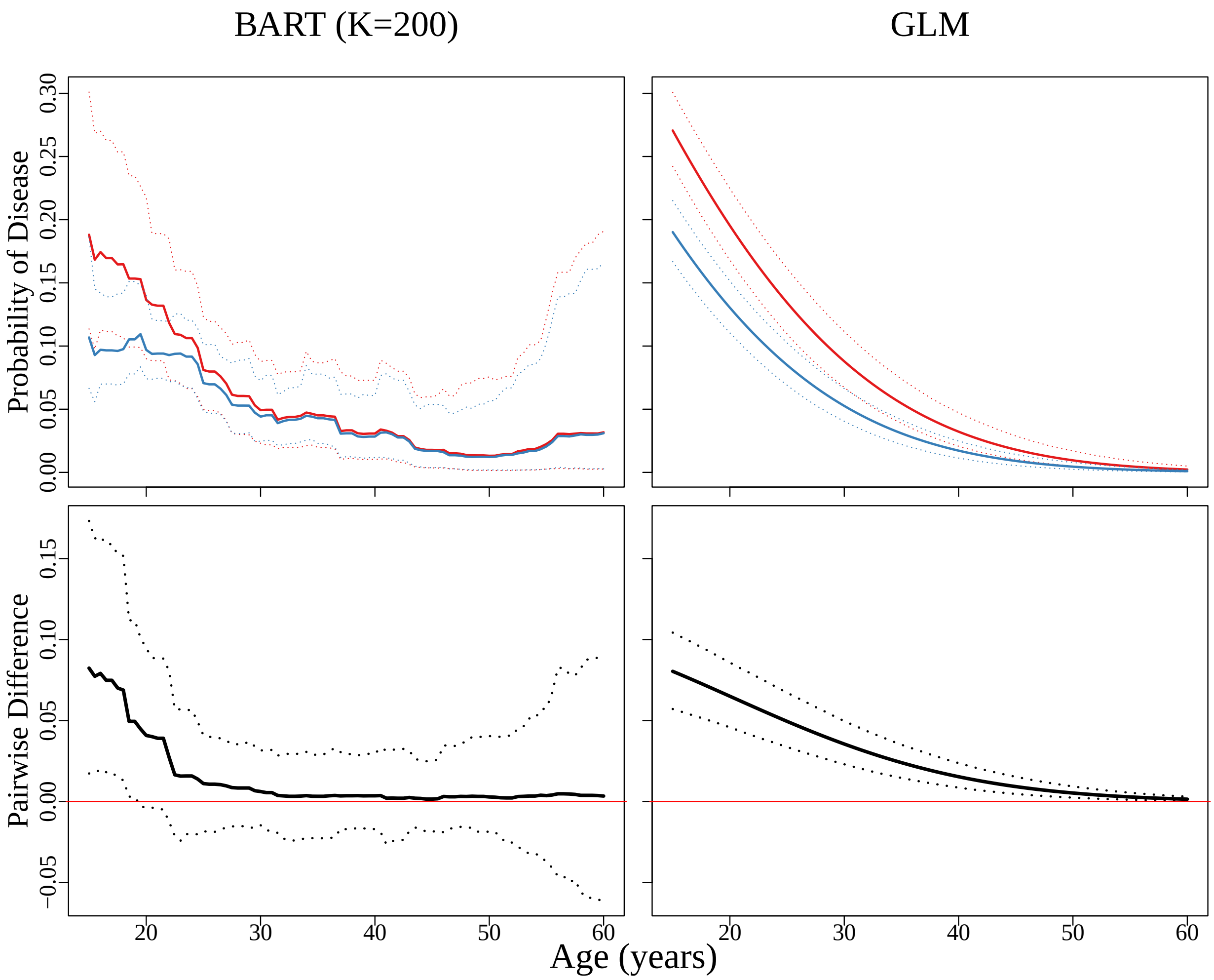

Figure F.2: Top: The solid red curve denotes posterior estimates of the probability of chlamydial disease for non-Caucasian females who reported having a new sexual partner ($x_{i2} = 0$, $x_{i3} = 1$). The solid blue curve denotes the same estimates for Caucasian females ($x_{i2} = 1$, $x_{i3} = 1$). Bottom: Difference in the estimated probabilities for the two cohorts in the top row. Pointwise 95% equal-tail credible bands are shown dotted. These figures were constructed by assuming noninformative priors for the AC2A accuracy probabilities and $K = 200$ trees.

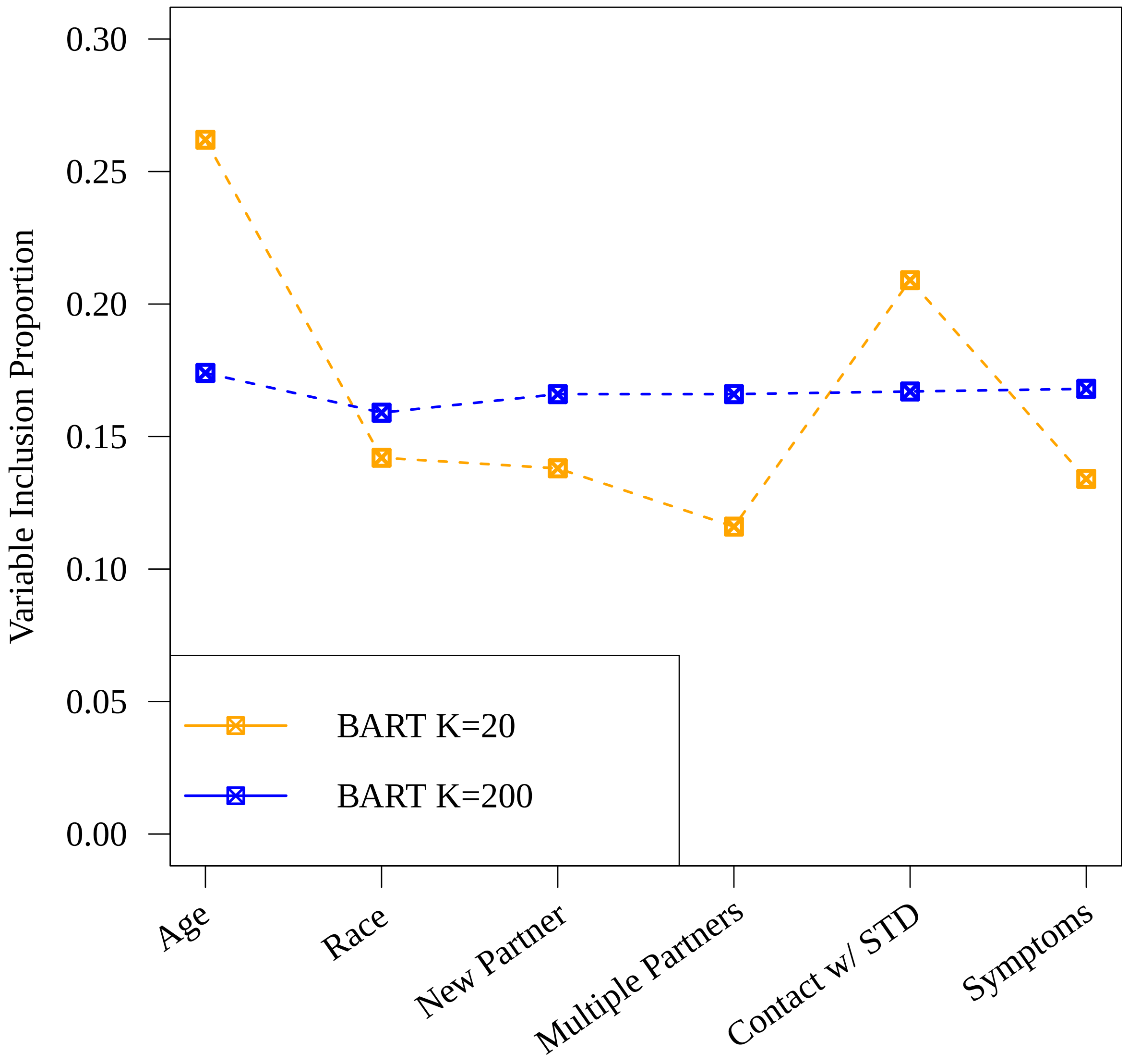

Figure F.3: Variable inclusion proportions using noninformative priors for the AC2A accuracy probabilities.

Table F.2: Estimation results for AC2A accuracy probabilities under noninformative priors. The posterior mean estimate (Est.), the estimated posterior standard deviation (ESE), and the 95% equal-tail credible interval (CI95) are shown. Results are shown for BART with $K = 20$ and $K = 200$ trees and the GLM fit (McMahan et al., 2017). All table values are shown using 2 decimal places.

		BART ($K = 20$)			BART ($K = 200$)			GLM		
		Est.	ESE	CI95	Est.	ESE	CI95	Est.	ESE	CI95
$S_{e(1)}$	Swab Individual	1.00	0.00	(0.99, 1.00)	1.00	0.00	(0.99, 1.00)	1.00	0.00	(0.99, 1.00)
$S_{e(2)}$	Urine Individual	0.85	0.08	(0.67, 0.99)	0.84	0.08	(0.65, 0.98)	0.57	0.06	(0.46, 0.68)
$S_{e(3)}$	Swab Pool	0.90	0.06	(0.79, 1.00)	0.89	0.07	(0.76, 0.99)	0.70	0.05	(0.62, 0.80)
$S_{p(1)}$	Swab Individual	0.98	0.01	(0.96, 0.99)	0.98	0.01	(0.96, 0.99)	0.99	0.01	(0.98, 1.00)
$S_{p(2)}$	Urine Individual	0.99	0.01	(0.97, 1.00)	0.99	0.01	(0.97, 1.00)	0.98	0.01	(0.97, 1.00)
$S_{p(3)}$	Swab Pool	1.00	0.00	(1.00, 1.00)	1.00	0.00	(1.00, 1.00)	1.00	0.00	(1.00, 1.00)

References

Chipman, H., George, E., and McCulloch, R. (2010). BART: Bayesian additive regression trees. *Annals of Applied Statistics* **4**, 266–298.

Chipman, H., George, E., and McCulloch, R. (1998). Bayesian CART model search. *Journal of the American Statistical Association* **93**, 935–948.

Gaydos, C., Quinn, T., Willis, D., Weissfeld, A., Hook, E., Martin, D., Ferrero, D., and Schachter, J. (2003). Performance of the APTIMA Combo 2 Assay for detection of *Chlamydia trachomatis* and *Neisseria gonorrhoeae* in female urine and endocervical swab specimens. *Journal of Clinical Microbiology* **41**, 304–309.

Hastie, T and Tibshirani, R. (2000). Bayesian backfitting. *Statistical Science* **15**, 196–213.

McMahan, C., Tebbs, J., Hanson, T., and Bilder (2017). Bayesian regression for group testing data. *Biometrics* **73**, 1443–1452.
